# Complex *N*-Glycans Are Important for Normal Fruit Ripening and Seed Development in Tomato

**DOI:** 10.3389/fpls.2021.635962

**Published:** 2021-03-09

**Authors:** Heidi Kaulfürst-Soboll, Melanie Mertens-Beer, Randolf Brehler, Markus Albert, Antje von Schaewen

**Affiliations:** ^1^Institute of Plant Biology and Biotechnology, University of Münster, Münster, Germany; ^2^Department of Dermatology, University of Münster, Münster, Germany; ^3^Molekulare Pflanzenphysiologie, Department Biologie, Friedrich-Alexander-Universität Erlangen-Nürnberg, Erlangen, Germany

**Keywords:** auxin-like effects, complex *N*-glycans, GNTI, hormone signaling, free *N*-glycans, fruit abscission, fruit ripening, MANII

## Abstract

Complex *N*-glycan modification of secretory glycoproteins in plants is still not well understood. Essential in animals, where a lack of complex *N*-glycans is embryo-lethal, their presence in plants seemed less relevant for a long time mostly because *Arabidopsis thaliana cgl1* mutants lacking *N*-acetyl-glucosaminyltransferase I (GNTI, the enzyme initiating complex *N*-glycan maturation in the Golgi apparatus) are viable and showed only minor impairments regarding stress tolerance or development. A different picture emerged when a rice (*Oryza sativa*) *gntI* T-DNA mutant was found to be unable to reach the reproductive stage. Here, we report on tomato (*Solanum lycopersicum*) lines that showed severe impairments upon two RNA interference (RNAi) approaches. Originally created to shed light on the role of *core* α1,3-fucose and β1,2-xylose residues in food allergy, plants with strongly reduced GNTI activity developed necrotic fruit-attached stalks and early fruit drop combined with patchy incomplete ripening. Correspondingly, semiquantitative RT-PCR of the abscission zone (az) revealed an increase of abscission markers. Also, *GNTI*-RNA interference (RNAi) plants were more susceptible to sporadic infection. To obtain vital tomatoes with comparable low allergenic potential, Golgi α-mannosidase II (MANII) was chosen as the second target. The resulting phenotypes were oppositional: MANII-reduced plants carried normal-looking fruits that remained attached for extended time without signs of necrosis. Fruits contained no or only few, but enlarged, seeds. Furthermore, leaves developed rolled-up rims simultaneously during the reproductive stage. Trials to cross MANII-reduced plants failed, while GNTI-reduced plants could be (back-)crossed, retaining their characteristic phenotype. This phenotype could not be overcome by ethephon or indole-3-acetic acid (IAA) application, but the latter was able to mimic patchy fruit ripening in wild-type. Phytohormones measured in leaves and 1-aminocyclopropane-1-carboxylic acid (ACC) contents in fruits showed no significant differences. Together, the findings hint at altered liberation/perception of protein-bound *N*-glycans, known to trigger auxin-like effects. Concomitantly, semiquantitative RT-PCR analysis revealed differences in auxin-responsive genes, indicating the importance of complex *N*-glycan modification for hormone signaling/crosstalk. Another possible role of altered glycoprotein life span seems subordinate, as concluded from transient expression of Arabidopsis KORRIGAN KOR1-GFP fusion proteins in RNAi plants of *Nicotiana benthamiana*. In summary, our analyses stress the importance of complex *N-*glycan maturation for normal plant responses, especially in fruit-bearing crops like tomato.

## Introduction

Plants are considered a safe and cost-effective way to produce therapeutic glycoproteins (Gomord et al., 2005; Schoberer and Strasser, 2018). *N*-glycosylation determines the physicochemical properties of glycoproteins, rendering this posttranslational modification indispensable for the functionality of most secretory proteins (Bosques et al., 2004; Gomord and Faye, 2004) and finally plant vitality (Lerouge et al., 1998; Strasser, 2016; Nagashima et al., 2018). The initial steps of *N*-glycan synthesis and protein attachment occur at/within the endoplasmic reticulum (ER). These basic steps are highly conserved in all eukaryotes due to folding assistance by lectin chaperones in the ER lumen (reviewed in Howell, 2013). Differences among taxa and species occur during *N*-glycan maturation, i.e., the conversion of ER-type *high mannose* to complex-type *N*-glycans in the Golgi apparatus (Varki et al., 2009). In animal and plant cells, in contrast to unicellular yeasts or microalgae (Mócsai et al., 2020), the initial *N*-glycan modifications in the Golgi stacks are similar (Varki, 2017). By the sequential action of several glycosyltransferases and glycosylhydrolases, *N*-glycans with less mannose but additional sugar moieties are formed (Kornfeld and Kornfeld, 1985). First, in the *cis*-Golgi, α-mannosidase I (Golgi MANI) removes terminal mannoses from the *high mannose* ER structure, resulting in Man5GlcNAc2∼Asn *N*-glycans (Kajiura et al., 2010). Then, *N*-acetyl-glucosaminyltransferase I (GNTI) adds an N-acetylglucosamine (GlcNAc) residue to the trimmed α1,3 arm (Harpaz and Schachter, 1980; Oppenheimer and Hill, 1981). Although it was reported that also abrogation of ER mannosidase I activity can lead to complex-type fucosylated and xylosylated *N*-glycans in the Golgi apparatus (Liebminger et al., 2009), no further *N*-glycan modification occurs when GNTI is missing. This was confirmed for the genetic model plant *Arabidopsis* by isolation and characterization of the first *complex glycan-less1* (*cgl1*) mutant lines (von Schaewen et al., 1993; Frank et al., 2008).

Downstream of GNTI, Golgi MANII removes two mannoses from the α1,6 arm (van den Elsen et al., 2001; Strasser et al., 2006; Shah et al., 2008), before *N*-acetyl-glucosaminyltransferase II (GNTII) can add one GlcNAc in place. Regardless of prior action of Golgi MANII (Kornfeld and Kornfeld, 1985; Lerouge et al., 1998; Bencúr et al., 2005; Strasser et al., 2006; Kaulfürst-Soboll et al., 2011b), xylose and *core* fucose residues may be added by β1,2-xylosyltransferase (XYLT) (Strasser et al., 2000) and α1,3-fucosyltransferase, the latter being encoded by two genes in *Arabidopsis*, FUCTa/FUT11 and FUCTb/FUT12 (Wilson et al., 2001; Bondili et al., 2006). Further decoration of the terminal GlcNAc residues leads to the formation of Lewis-a epitopes (Léonard et al., 2002) *via* sequential action of β1,3-galactosyltransferase (GALT1) and α1,4-fucosyltransferase FUCTc/FUT13 (in *Arabidopsis*; Strasser et al., 2007). This bulky modification seems to occur on glycoproteins destined for the plasma membrane/apoplast, but not on those targeted to the tonoplast/vacuole (Takahashi et al., 1986; Fitchette-Lainé et al., 1997). Abundance of Lewis-a epitopes varies among different plant species and tissues (Wilson et al., 2001; Strasser et al., 2007).

Complex *N*-glycans of plant origin differ from those in mammals by their *core* α1,3-fucose linkage (also present in insects and certain helminths) and β1,2-xylose attached to the branching mannose (also found in helminths and some snails). In the medical literature, especially in the field of allergy, these epitopes are known as cross-reactive carbohydrate determinants (CCDs). Based on their ability to be bound by specific immunoglobulin E (IgE) antibodies, regardless of the origin, they appear to react similarly to pan-allergens, although in this case without clinical symptoms (Aalberse et al., 1981; reviewed by Altmann, 2007). However, crops are an essential part of the human diet, and oral intake/digestion of plant glycoproteins is usually well tolerated and causes no problems. Nevertheless, CCD-specific IgE antibodies are especially abundant in humans with pollen and/or hymenoptera (bee/wasp) allergy, where they are able to activate basophilic granulocytes (Mertens et al., 2010; Kaulfürst-Soboll et al., 2011a; and the references cited therein). Immunogenicity becomes more problematic with respect to the parenteral administration of glycoproteins containing non-human carbohydrate structures (Gomord et al., 2005; Walsh and Jefferis, 2006). This became evident when patients responded with severe acute symptoms to the injection of cetuximab (Erbitux^®^), a chimeric mouse–human monoclonal antibody carrying the non-primate glycan epitope Gal-α1,3-Gal of mouse, common with meat from cows and pigs, but also present in cats, dogs, and rats (Chung et al., 2008).

In order to overcome the risk of hypersensitive immune reactions connected with plant-made pharmaceuticals (PMPs), much effort went into altering the modification of *N*-glycans in different plant hosts (Saint-Jore-Dupas et al., 2004). First, retention within the ER was tested as a solution to protect glycoproteins from receiving *core* fucose or xylose residues in the Golgi. Fusion of the amino-acid motif H/KDEL to the C-terminus was found sufficient for ER retention of candidate proteins (Denecke et al., 1992) by carrying mostly *high mannose N*-glycans with eight to nine Man residues (Pagny et al., 2000). But since retention of heterologous proteins in the ER depends on the efficiency of the retrieval machinery (bringing proteins back from the Golgi apparatus), some glycoproteins were still found to be modified with *complex-type N*-glycans (e.g., cell wall invertase; Pagny et al., 2000). Nevertheless, plant-made antiviral antibodies were shown to be as functional as their mammalian counterparts- yet, with shorter half-life due to a higher clearance rate by mannose receptors in mammals (Engering et al., 1997; Ko et al., 2003).

Another approach to reduce the immunogenic potential of PMPs is the knockout or silencing of plant glycosyltransferases or glycosylhydrolases. Besides eliminating *core* fucose and xylose by a mutant combination or the engineering of *Nicotiana benthamiana* plants (Strasser et al., 2004, 2008), *Arabidopsis complex glycan-less1* (*cgl1* = *gntI*) produces mostly glycoproteins with mannose-terminating *N*-glycans of Man5GlcNAc2∼Asn structure (von Schaewen et al., 1993). Feasibility to produce an active human enzyme for therapeutic treatment of a congenital genetic disorder was proven by heterologous expression of glucocerebosidase in *Arabidopsis cgl1* seeds (Downing et al., 2006). However, *Arabidopsis* is a small weed and usually not well suited for obtaining high yields, therefore gene-silencing approaches were pursued to suppress GNTI activity in crops. First approaches demonstrated moderate success: *GNTI* silencing by antisense approaches in potato and tobacco resulted in a clear, age-dependent reduction of the complex *N*-glycan pattern (Wenderoth and von Schaewen, 2000), but sometimes without visible changes (Strasser et al., 2004). Better suppression was later obtained by double strand-based RNA interference (dsRNAi) in potato and tomato (Kaulfürst-Soboll et al., 2011a).

Although knockout or silencing of *GNTI* is an efficient tool to reduce the allergenic potential of plant-specific *N*-glycan epitopes, it turned out to be disadvantageous for the plant itself. In *Arabidopsis*, lack of/reduced complex *N*-glycan formation resulted in increased salt sensitivity, showing as compromised root growth (Frank et al., 2008; Kang et al., 2008). Besides, an extended flowering period, reported by Boyes et al. (2001), and susceptibility to sporadic pathogen attack, already observed for *Arabidopsis cgl1* (von Schaewen et al., 1993), was later also found for tomato *GNTI*-RNAi plants (Kaulfürst-Soboll et al., 2011a). More drastic effects were observed in rice *gntI* T-DNA plants that suffered from severe developmental problems and failed to reach the reproductive stage (Fanata et al., 2013).

So far only observed in tomato, stalk-associated fruit parts of *GNTI*-RNAi plants turned brown during ripening, leading to premature fruit drop (Kaulfürst-Soboll et al., 2011a). Interestingly, in *Arabidopsis hybrid glycosylation1* (*hgl1* = *manII*) mutants, both *core* fucose and xylose residues are masked from full immunogenic recognition (Kaulfürst-Soboll et al., 2011b); therefore, we chose the next enzyme in line, Golgi mannosidase II (MANII), as alternative for reducing the immunogenic potential of tomato fruits. To examine whether silencing of Golgi *MANII* may be better tolerated or provokes a similar ripening phenotype as observed upon *GNTI* silencing, we generated stable Micro-Tom *MANII*-RNAi lines using two different dsRNAi constructs. Comparative analyses with wild-type and *GNTI*-RNAi lines grown in parallel are presented here.

## Materials and Methods

### Plant Material

The *Solanum lycopersicum* variety Moneymaker dwarf cultivar Micro-Tom (Scott and Harbaugh, 1989) is defective in genes of brassinosteroid metabolism (which indirectly influences responsiveness to gibberellin) and carries a mutation in the self-pruning (*sp*) gene that leads to a determinate phenotype and another still uncharacterized mutation. In addition, supplemental resistance genes are present, e.g., for *Fusarium* wilt race 1, gray leafspot, and *Cladosporium fulvum* (Scott and Harbaugh, 1989; Martí et al., 2006). Still, Micro-Tom is considered a suitable model for the analyses of hormonal regulations in fruit development (Meissner et al., 1997; Eyal and Levy, 2002; Martí et al., 2006; Serrani et al., 2007). Transformation of Micro-Tom cotyledons by Agrobacteria cocultivation in tissue culture and further propagation were done as described previously (Kaulfürst-Soboll et al., 2011a). Tomato plants in soil (for perennials) were regularly watered with 0.4 ppm CALCINIT (93.5% NO_3_^–^, 6.5% NH_4_^+^, 27% Ca^2+^, water-soluble) and 0.2 ppm HaKaPhos basis3 (COMPO, Germany). *N. benthamiana* plants were watered with complete fertilizer HaKaPhos plus (52% NO_3_^–^, 48% NH_4_^+^ in N14+P5+K24, COMPO, Germany). In the greenhouse, plants received additional illumination (long day regime: 16-h light/8-h darkness at 20–21 to 24–25°C). When about 3 weeks old, *N. benthamiana* plants were transferred to the lab for agroinfiltration.

### Cloning of RNAi Constructs and Generation of Stably Transformed Plants

Tomato *MANII*-RNAi transformants were created analogous to the *GNTI*-RNAi transformants described previously (Kaulfürst-Soboll et al., 2011a). A circa 400-bp fragment of the gene coding for Golgi MANII was obtained *via* RT-PCR from total leaf RNA of Micro-Tom and inserted twice *via* compatible restriction sites into pUC-RNAi (SalI/BamHI and/or XhoI/BglII; Supplementary Figure S1), flanking the first intron of potato GA20 oxidase (Chen et al., 2003). One construct was assembled in sense-intron-antisense orientation, and another in antisense-intron-sense orientation, using the primers given in Supplementary Table S1. The two assemblies were inserted *via* PstI into SdaI opened plant-expression vector pBinAR (HygR) behind the constitutive CaMV 35S promoter (Höfgen and Willmitzer, 1990), introduced into Agrobacteria and used to transform wild-type Micro-Tom.

The sense-intron-antisense *MANII*-RNAi construct was also used to transform *N. benthamiana* using a tissue-culture protocol for *Nicotiana tabacum* (Faske et al., 1997). For RNAi suppression of *GNTI*, another construct was used (Supplementary Figure S1). A similar region as chosen for tomato *GNTI*-RNAi (Kaulfürst-Soboll et al., 2011a) was amplified from *N. tabacum* Samsun NN clone A9 (Wenderoth and von Schaewen, 2000) using the same primers as for tomato *GNTI*-RNAi and inserted twice into vector pUC-RNAi in sense-intron-antisense orientation. The assembly was first inserted *via* PstI into the SdaI site between the CaMV 35S promoter and octopine synthase terminator. The entire expression cassette was released by EcoRI and SspI and inserted into EcoRI/SnabI-opened binary vector pDE1001 (KanR). All constructs were verified by restriction analyses/sequencing prior to *Agrobacteria*-mediated plant transformation.

### Immunoblot Analyses and Peptide:N-Glycosidase F Treatment

Immunoblot analyses and peptide:N-glycosidase F (PNGase F, NEB) treatment were done with fruit extracts as described earlier (Kaulfürst-Soboll et al., 2011a, b) using α-PHA-L rabbit antiserum for routine cgly detection. α-vINV rabbit antiserum served as the marker of the ripening stage-dependent vacuolar invertase (vINV), concanavalin A (ConA) for detection of mostly mannose-terminating *N*-glycans, and selected potato-tomato allergic patient sera for the detection of cross-reactive carbohydrate determinants (CCDs), all characterized in Kaulfürst-Soboll et al. (2011a).

### Basophil Activation Test

The basophil activation tests (BATs) were performed as described earlier (Kaulfürst-Soboll et al., 2011a) with heparinized whole blood from potato–tomato allergic patients using flow cytometry.

### Root Growth Analyses

Root growth responses of tomato seedlings to salt were analyzed as described for *Arabidopsis* in Kang et al. (2008). Prior to germination, seeds were surface-sterilized in 50-ml tubes, 1 × 10 min with 4% sodium hypochloride (NaOCl) and washed 2 × 10 min with sterile ddH_2_O. Seedlings were kept on normal 3MS medium (complete Murashige & Skoog with vitamins, 3% sucrose, pH 6.1) before transfer to vertical agar (1.5%) plates of the same medium with salt (100 mM NaCl).

### Fruit Picking, Seed Harvesting, and Hormone Treatments

To determine how tightly tomato fruits are attached to their mother plants, the force needed to pick a fruit was measured. Three-month-old Micro-Tom plants grown in the greenhouse were used for the experiments. Fruits of similar developmental stage were pulled in longitudinal direction, and the force was recorded with a computer-assisted dynamometer.

Tomato seeds of ripe fruits were acid-washed in 1 N HCl for several hours and then extensively washed with tap water before letting them dry on filter paper. Dried seeds were stored for several months before using them for seed weight determinations.

Ethephon treatment of fruits was essentially done as described in Mizrahi et al. (1976). Fruits were dipped for 1 min into an aqueous solution [2% ethephon in 10 mM 2-(N-morpholino)ethanesulfonic acid (MES), pH 5] and left to dry. Auxin was applied with a syringe. Prior to infiltration, relief holes were pinched into each fruit with the help of the injection needle. Then, ca. 100–300 μl of indole-3-acetic acid (IAA) (100 μM IAA in 10 mM MES, pH 5) was slowly injected, with one repetition on the following day. Buffered water served as control.

### Phytohormone Measurements of Tomato Leaves

Leaves were harvested from 4–5-week-old tomato plants for analysis of salicylic acid (SA), auxin (IAA), abscisic acid (ABA), and jasmonic acid (JA). After grinding under liquid N_2_ to fine powder, 200 mg was either stored at −80°C or directly used for extraction. Extraction of the free analytes was carried out with 1.5 ml ethyl acetate, containing 0.1% (v/v) formic acid and the internal standards 3-hydroxybenzoeic acid (3HOBA), dihydro-jasmonic acid, and indole-5-carboxylic acid (5IFA) (50 ng ml^–1^). Samples were incubated at 28°C for 60 min after a 10-min sonification step in an ultrasonic bath. After centrifugation at 18,500 *g*, 1.2 ml of the supernatant was transferred to a new tube. The ethyl acetate was removed to dryness in a gentle stream of N_2_. Derivatization was performed with 70 μl of a 1:1 mixture of methanol:trimethylsilyldiazomethane (TMSDM; 2 M in diethyl ether) for 20 min at 25°C. Determination of the analytes in 1 μl injected volume was performed by gas chromatography/mass spectrometry (GC/MS) (Shimadzu TQ8040) using splitless injection mode and an SH-Rxi-17SIL-MS column (30 m, 0.25 mm internal diameter, 0.25 μm film, RESTEK GmbH, Germany). The GC oven temperature was held at 70°C for 5 min, then ramped at 15°C min^–1^ to 270°C, then ramped at 75°C min^–1^ to 280°C, and afterward held for an additional 10 min at 280°C. Helium was used as the carrier gas with a flow rate of 1 ml min^–1^. The mass spectrometer was operated in electron impact ionization (EI) and multiple reaction monitoring (MRM) mode, also described in Wan et al. (2019).

For measurements of leaf-emitted ethylene, leaves of 4–6-week-old tomato plants were cut in small squares (3 × 3 mm, using sizers). Leaf pieces were floated on water in a Petri dish overnight (at room temperature) for recovery, before three (each) were transferred into a 6-ml glass reaction tube containing 500 μl water (dd): without treatment, after wounding (3× squeezing with a spatula), or addition of 1 μM elicitor (flg22; Felix et al., 1999; Crip21; Hegenauer et al., 2020). Vials were sealed with rubber plugs and placed on a horizontal shaker (80–100 r/min) at room temperature for 3 h. Of the gas phase, 1 ml was analyzed with a GC-flame ionization detector (FID) (Shimadzu, GC-2014, glass column 3 mm × 1.6 m with Al_2_O_3_).

### 1-Aminocyclopropane-1-Carboxylic Acid Measurements of Tomato Fruits

Ethylene was chemically released from 1-aminocyclopropane-1-carboxylic acid (ACC) as described by Lizada and Yang (1979). Frozen tomatoes were thawed, cut in half, and photographed. One fruit half was crushed with a mortar and pestle in liquid nitrogen. Then, 50% methanol solution (10 ml g^–1^ fresh weight) was added to the powder. The homogenate was heated to 60°C for 15 min. The suspension was distributed to the test tubes (1 ml per tube). HgCl_2_ was added to a final concentration of 100 μM, sodium hydroxide (NaOH) was added to a final concentration of 600 mM, and hypochlorite (NaOCl) to 0.35% v/v. Tubes were immediately sealed with rubber plugs and incubated on ice for 20 min. Afterward, 1 ml gas phase was analyzed with a GC-FID (Shimadzu, GC-2014, glass column 3 mm × 1.6 m with Al_2_O_3_).

### Semiquantitative RT-PCR

Several fruit-bearing pedicels of the same ripening stage [extending 3 mm to both sides of the abscission zone (az)] were harvested and snap frozen in liquid nitrogen. Total RNA was extracted according to Sokolovsky et al. (1990) and treated with RNase-free DNase I. First-strand cDNA was synthesized from 2 μg total RNA in a volume of 40 μl using oligo-dT (18-mer), RiboLock RNase inhibitor (Thermo Scientific), and RevertAid^TM^ H Minus M-MuLV Reverse Transcriptase (Fermentas). For PCR with Taq-DNA Polymerase (Biozyme), 1-μl aliquots and gene-specific primer pairs were used at optimized annealing temperatures and the cycles indicated in Supplementary Table S1.

### KOR1-GFP Cloning and Expression Analyses

Cloning of the KOR1-GFP construct was done by restriction digest of pGFP2ΔNco-KOR1 (Rips et al., 2014) and insertion *via* XbaI/EcoRI sites 5’ of the GFP reporter in pGPTVII.Hyg (Walter et al., 2004). The final construct pGPTVII.Hyg:KOR1-GFP was used to transform Agrobacteria (strain GV2260). Leaves of 4-week-old *N. benthamiana* plants (wild-type, *GNTI*-RNAi, and *MANII*-RNAi) were co-infiltrated with silencing suppressor strain 19K and placed for 24 h in the dark before transfer to constant light. Leaf samples were checked for GFP signals [confocal laser scanning microscopy (CLSM), Leica SP5] and infiltrated with 10 μM tunicamycin as described in Frank et al. (2008).

### Immunoblot Analyses of *Agrobacterium*-Infiltrated Leaves

For each sample, two leaf discs each were excised with a cork borer (∅ 5 mm) and frozen in liquid nitrogen. Protein extraction and immunoblot analyses were essentially done as described earlier (Frank et al., 2008). A first extraction step was conducted without sodium dodecyl sulfate (SDS) to yield a more concentrated extract in the second step with SDS. The first extraction was done in 50 mM HEPES-NaOH, pH 7.5, 250 mM NaCl, 2 mM sodium pyrosulfite, 1 mM ethylenediaminetetraacetic acid (EDTA), 1 mM Pefabloc SC (Applichem), proteinase inhibitor cocktail for use with plant extracts (Sigma, 1 μl per 30 mg fresh weight), and 2% β-mercaptoethanol. Then, 1% [w/v] insoluble polyvinylpolypyrollidone (PVPP, Sigma) and 2% [w/v] inulin (Merck) were added to the buffer and thoroughly mixed before distribution to the samples. After centrifugation for 10 min in a tabletop centrifuge (4°C), the obtained pellet was extracted with 80 μl of the buffer containing 0.6% [w/v] SDS (w/o PVPP or inulin). SDS-loading buffer was added to 30 μl of the cleared supernatants (4°C), boiled for 10 min, and loaded onto a 6% polyacrylamide gel for SDS-polyacrylamide gel electrophoresis (PAGE) followed by immunoblot analysis.

## Results

### Complex *N*-Glycan Recognition Is Strongly Reduced in Tomato *MANII*-RNAi Plants

**FIGURE 1:**
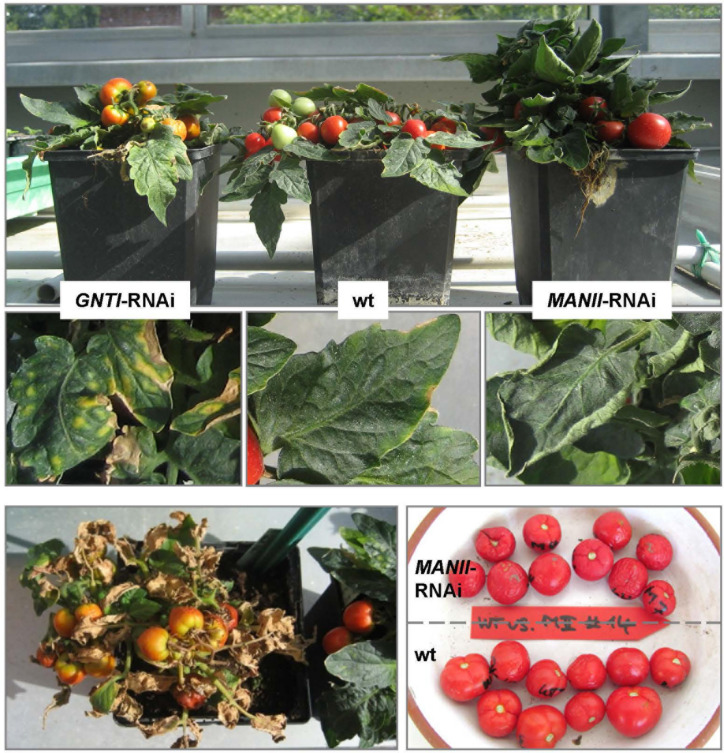
*GNTI*- and *MANII*-RNAi plants show opposite growth phenotypes. Micro-Tom wild-type (wt) next to GNTI-RNAi (#20) and MANII-RNAi (#14) plants in the greenhouse. Around the age of 4 months, leaves of wt plants started to develop yellow rims (center), while premature senescence was visible for GNTI-RNAi plants, with yellow to necrotic leaf sectors (center left). At this stage, MANII-RNAi plants showed no signs of senescence, but often rolled-up leaf rims (center right). When cut back, GNTI-RNAi plants were able to grow new green sprouts (bottom left). Leaves and fruits of MANII-RNAi plants had a darker, matte appearance (center and bottom right). For details on plant growth and flower and fruit phenotypes, see Supplementary Figure S3.

According to analyses of *Arabidopsis hgl1* mutants (Kaulfürst-Soboll et al., 2011b), suppression of Golgi MANII activity should result in reduced complex *N*-glycan (cgly) recognition. As previously done for *GNTI* (Kaulfürst-Soboll et al., 2011a), *MANII* was silenced in the tomato Moneymaker dwarf variety Micro-Tom *via* a stable dsRNAi approach. In addition to the usual sense-intron-antisense orientation, also the reciprocal antisense-intron-sense orientation was tested (Supplementary Figure S1A). Compared to *GNTI*-RNAi, many more regenerants were obtained for both *MANII*-RNAi constructs. Both leaf and fruit extracts of the primary transformants were tested for reduced cgly patterns on immunoblots using a rabbit α-cgly antiserum (α-PHA-L; Laurière et al., 1989), mostly containing xylose-specific but also *core* fucose-specific antibodies. Independent of the sense and antisense orientation, strong suppression of cgly recognition was achieved by both *MANII*-RNAi constructs similarly to *GNTI*-RNAi (Supplementary Figures S1B,C). Selected transformants were propagated further (Figure 1), and leaf extracts were analyzed in parallel to a *GNTI*-RNAi (T3) plant, side by side with *Arabidopsis* cgly mutants (Supplementary Figure S2), using α-PHA-L and α-HRP antisera with slightly different binding affinities (Kaulfürst-Soboll et al., 2011b). Strongly suppressed plants among the tested primary transformants were about 50% for both *MANII*-RNAi constructs compared to only 7% previously obtained for the *GNTI*-RNAi lines. Of initially 91 independent *GNTI*-RNAi transformants, only lines #20 and #45 could be used for the analyses because among six substantially silenced T0 plants, one of the strongest candidates (#91) was lost upon transfer from tissue culture to soil. Of note, using a fruit-specific promoter (B33, driving the same *GNTI*-RNAi cassette), not a single silenced plant was obtained, although proven effective for *vINV*-RNAi suppression (Kaulfürst-Soboll et al., 2011a). For *MANII*-RNAi, lines #5 (producing only few seeds), #6, #11, #13, #14, #15, and #18 were propagated further (Supplementary Figures S3A,B), with lines #11, #14, and #18 used for most of the experiments. Their analyses showed that *MANII*-RNAi silencing in tomato is stable, resulting in low cgly recognition, comparable to that of the *Arabidopsis hgl1* (*manII*) mutant.

### Tomato *MANII*-RNAi Plants Show Subtle Growth Phenotypes

When grown in the greenhouse next to wild-type and *GNTI*-RNAi, *MANII*-RNAi plants appeared more vigorous (Figure 1, top) with delayed leaf senescence. In case of sporadic pathogen attack (or abiotic stress without clear specification), *MANII*-RNAi plants performed like wild-type, while *GNTI*-RNAi plants were highly susceptible. Especially stems and flowers of *MANII*-RNAi plants were larger, and fruits developed slightly differently (Supplementary Figure S3C). Another specific phenomenon of *MANII*-RNAi plants was that leaves had a matte appearance, which was also true for fruits (Figure 1 and Supplementary Figure S3A, bottom), and sometimes displayed less complex leaf shapes. Furthermore, they rolled up their rims, indicating enhanced growth of the lower and/or inhibited growth of the upper side. This happened synchronously (from one day to the next) and was only observed during the fruit-bearing period, pointing to involvement of a volatile phytohormone (perhaps ethylene as antagonist of auxin) without affecting the other genotypes cultivated in parallel. When cut back, *GNTI*-RNAi plants showing early senescence/necrosis were able to regenerate new green sprouts (Figure 1, bottom left). Interestingly, root growth of *MANII*-RNAi seedlings was more tolerant to salt (100 mM NaCl), whereas *GNTI*-RNAi seemed not affected (Supplementary Figure S4).

### Extent of Complex Glycan Suppression During Fruit Ripening

**FIGURE 2:**
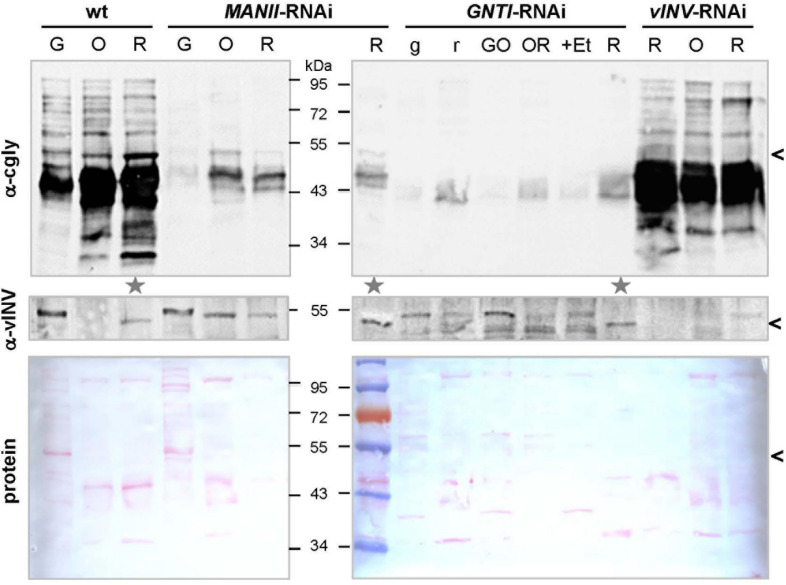
Detection of complex *N*-glycosylated proteins during tomato fruit ripening. Blots of fruit extracts from Micro-Tom wild-type (wt), *MANII*-RNAi (#14), *GNTI*-RNAi (#20), and vInv-RNAi (#9) plants of different ripeness (G, green; O, orange; R, red fruits, with lowercase letters indicating fruit parts; +Et, ethephon treatment) were developed with antisera specific for vacuolar invertase (α-vINV, top) or complex *N*-glycans (α-cgly, PHA-L, center). The latter mainly recognizes β1,2-xylose and to a lesser extent *core* α1,3-fucoses (independent antibodies). The Ponceau S-stained blots (protein) are shown as loading reference. Since vacuolar invertase is induced during tomato fruit ripening (migrating ∼50 kDa, avoid in *vINV*-RNAi), its presence was used as the marker to select fruit extracts of similar ripeness for further use (gray stars). Note that vINV (carrying four complex *N*-glycans in wild-type) is not the most abundant glycoprotein in red fruits (black arrowheads). Proteins decorated with complex *N*-glycans accumulate during ripening in wild-type fruits (left), which is markedly reduced in the *MANII*-RNAi and *GNTI*-RNAi lines. Apparent molecular masses are indicated in kDa (PageRuler Prestained Protein Ladder, Fermentas).

The abundance of glycoproteins in tomato fruits increases upon ripening (Priem and Gross, 1992), without structural differences between *N*-glycans of green and red fruits (Zeleny et al., 1999). To demonstrate this for wild-type and evaluate the extent of cgly recognition in *MANII*-RNAi versus *GNTI*-RNAi plants, fruits of different ripening stages were harvested for immunoblot analyses. Because the patchiness of *GNTI*-RNAi fruits matched the 35S-promoter expression pattern (as visualized by β-glucuronidase GUS fusions in ripe fruits; Moon and Callahan, 2004), green and red parts were separated. Although fruit color is a good indicator of ripeness (indicated by letters in Figure 2), vINV was used as an additional marker because mRNA levels are induced by ethylene and dramatically increase with fruit ripening (Klann et al., 1993; Alba et al., 2005). Moreover, the protein was undetectable in *vINV*-RNAi fruits (Kaulfürst-Soboll et al., 2011a). Red fruits with comparable amounts of mature vINV signal in wild-type, *GNTI*-RNAi, and *MANII*-RNAi extracts were marked for further analyses (Figure 2, gray stars). As expected, the complex glycan pattern increased upon fruit ripening in wild-type (α-cgly) but was significantly reduced by *GNTI*-RNAi and *MANII*-RNAi silencing (although remaining bands were detected for both). To determine the extent of cgly reduction in ripe fruits more accurately, selected extracts were serially diluted prior to SDS-PAGE. Blots were developed with anti-complex glycan antiserum (α-cgly; Supplementary Figure S5) and also by affinoblotting with the lectin ConA (Faye and Chrispeels, 1985; Brewer and Bhattacharyya, 1986). Binding of cgly-specific antibodies was similarly reduced in *GNTI*-RNAi and *MANII*-RNAi fruit extracts, about 16–32 times compared to wild-type. Inversely, ConA labeling increased to the same extent in both *GNTI*-RNAi and *MANII*-RNAi extracts, as shown for *Arabidopsis gntI* mutant *cgl1* (von Schaewen et al., 1993).

**FIGURE 3:**
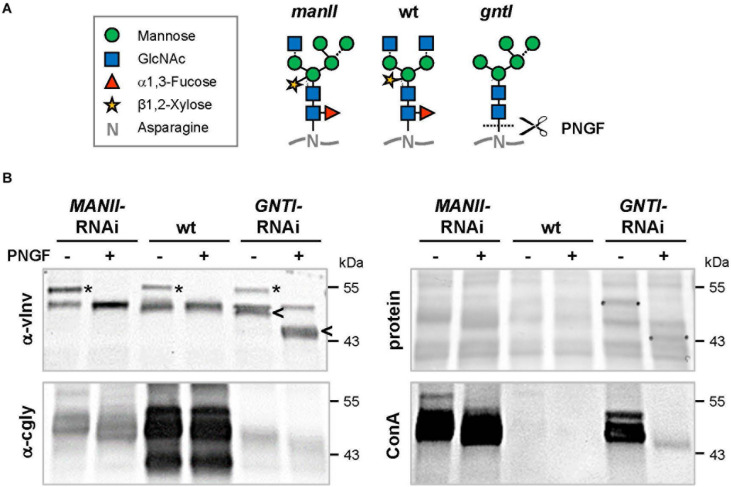
Peptide:N-glycosidase F (PNGase F) treatment verifies the presence of *core* fucoses on *MANII*-RNAi glycans. **(A)** *N*-glycan structures (www.proglycan.com) that may be produced in wild-type (wt), *manII*, and *gntI* mutant plants (known as *hgl1* and *cgl1* in *Arabidopsis*, respectively) with indicated potential release by PNGase F. This endoglycosidase can cleave asparagine-bound *N*-glycans when *core*α1,3-fucose is missing. In the cell wall or vacuole, terminal sugar residues can be liberated by hexosaminidases (GlcNAc) and α-mannosidases (dotted lines). **(B)** Immunoblots of untreated (-) and PNGase F-treated (+) fruit extracts from *MANII*-RNAi (#14), Micro-Tom wild-type (wt), and *GNTI-*RNAi (#20) plants were developed with α-vINV, α-cgly (α-PHA-L), or the lectin ConA (detecting mannose-terminating *N*-glycans). The Ponceau S-stained blot (protein) is shown as the loading reference. In *GNTI-*RNAi, PNGase-F treatment results in a shift of vacuolar invertase (vINV, arrowheads) and loss of ConA binding. This is not the case for *MANII*-RNAi or wt samples, confirming the presence of *core* fucoses on most glycoproteins. Note that the upper band detected by α-vINV (stars) shifts in all PNGase F-treated extracts (protein with high mannose precursor), indicating completeness of the enzyme treatment. Apparent molecular masses are indicated in kDa (PageRuler Prestained Protein Ladder, Fermentas).

In Kaulfürst-Soboll et al. (2011a), GNTI reduction in fruits was verified by immunoblot analyses of protein extracts treated with PNGase F, an enzyme that releases asparagine-bound *N*-glycans when α1,3-fucose is missing (Tretter et al., 1991). Glycoproteins of *GNTI*-RNAi plants (or *gntI* mutants) are PNGase F-sensitive (Figure 3A), as indicated by the vINV shift (four complex *N*-glycans in wild-type) and inverse loss of ConA binding to most glycoproteins (Figure 3B). No shifts were detected for wild-type and only minor ones for *MANII*-RNAi extracts (compare α-cgly to ConA), confirming the presence of *core* fucoses on most protein-bound *N*-glycans of *MANII*-RNAi fruits, as previously shown for the *Arabidopsis manII* mutant *hgl1* (Strasser et al., 2006; Kaulfürst-Soboll et al., 2011b).

### *MANII*-RNAi Silencing Reduces the Immunogenic Potential of Tomato Fruits

**FIGURE 4:**
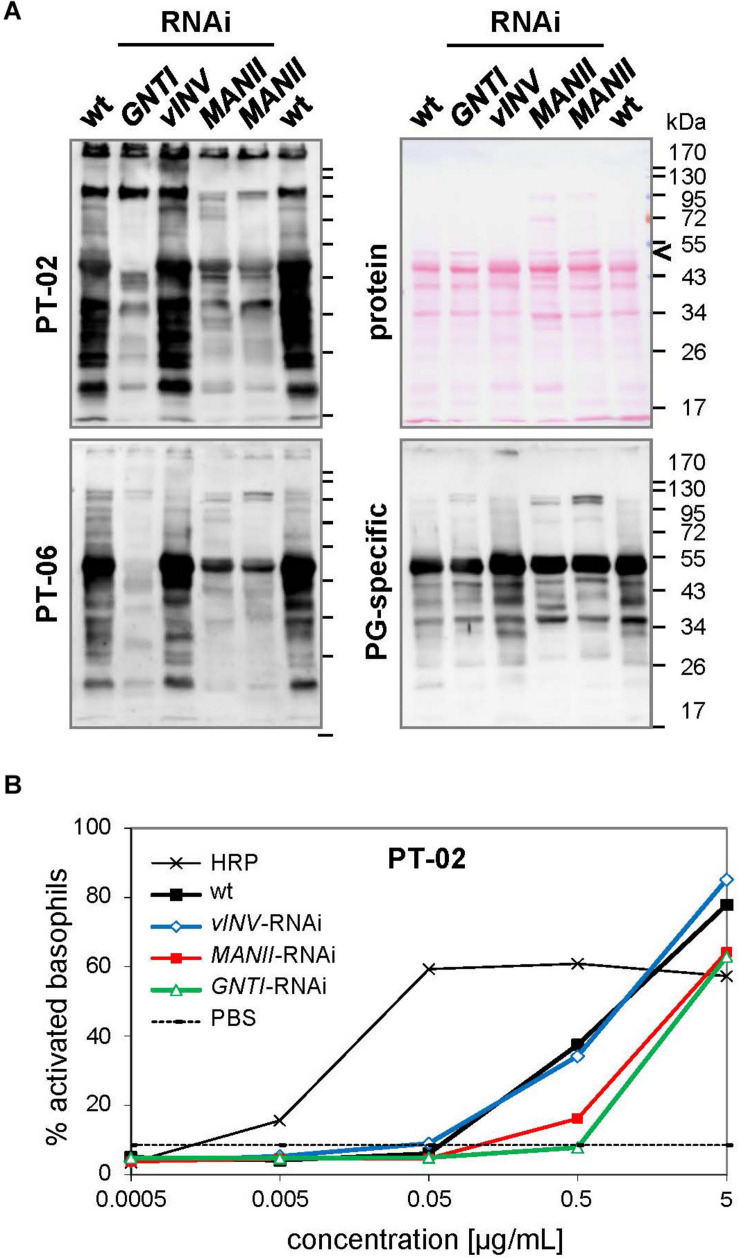
Patient sera reveal reduced cross-reactive carbohydrate determinants (CCD) binding to *MANII*-RNAi fruit extracts. **(A)** Immunoblots were prepared with extracts of Micro-Tom wild-type (wt) and RNAi-silenced fruits (*GNTI*-RNAi #20; vINV #9; *MANII*-RNAi lines #11 and #14). CCD-positive sera of potato/tomato-allergic patients PT-02 and PT-06 (Kaulfürst-Soboll et al., 2011a) were used for the detection of CCD-specific IgE (sIgE) antibodies. A serum of a non-allergic individual, also containing sIgE for polygalacturonase 2A (46 kDa, a major glycoprotein of tomato fruits), but only minor CCD-sIgE, was included as control. The Ponceau S-stained blot (protein) is shown as the loading reference. The black arrowhead points to vacuolar invertase (vINV, 52 kDa), which was not detected in *vINV*-RNAi fruits. Note that the CCD-positive patient sera (left) show strong binding to wt and *vINV*-RNAi, but reduced binding to *GNTI*-RNAi and *MANII*-RNAi extracts. Apparent molecular masses are indicated in kDa (PageRuler Prestained Protein Ladder, Fermentas). **(B)** Whole blood of patient PT-02 was used for a basophil activation test (BAT) with the indicated fruit extracts, demonstrating about 10 times reduced stimulation by *GNTI*-RNAi and also *MANII*-RNAi. Horseradish peroxidase (HRP; vacuolar glycoprotein with nine complex *N*-glycans) in PBS served as a positive control for CCD-dependent basophil stimulation and PBS as negative control.

From the analyses of *Arabidopsis hgl1* mutants, MANII emerged as an alternative target for lowering the immunogenic potential of plant-derived glycoproteins (Kaulfürst-Soboll et al., 2011b). This was proven first by Western blot analysis using CCD-positive sera of two potato/tomato-allergic patients that contain CCD-specific (s)IgE antibodies (mediators of immediate-type allergic reactions). Patient sera, mainly recognizing *core* fucose (PT-02) or xylose epitopes (PT-06; Kaulfürst-Soboll et al., 2011a; Kaulfürst-Soboll et al., 2011b), showed markedly reduced binding to *MANII*-RNAi fruit extracts. Of note, binding was comparable to *GNTI*-RNAi (Figure 4A), whereas binding to *vINV*-RNAi extracts resembled wild-type, as reported earlier (Kaulfürst-Soboll et al., 2011a). To confirm similar peptide-epitope composition, the serum of a latex-allergic patient that strongly binds to highly abundant tomato polygalacturonase 2A (PG), confirmed with the recombinant protein expressed in *Escherichia coli* (not shown), was included.

To further investigate whether *MANII*-RNAi suppression in tomato also results in lower effector-cell triggering, as previously shown for *GNTI*-RNAi (Kaulfürst-Soboll et al., 2011a), an *ex vivo* BAT was performed with whole blood of PT-02 and native tomato fruit extracts. Basophils of this potato/tomato-allergic patient were strongly activated by horseradish peroxidase (HRP; nine complex *N*-glycans), routinely used to demonstrate activation by plant-derived CCD epitopes (Figure 4B). Activation by wild-type or *vINV*-RNAi extracts was similar but required about 10 times higher concentration of the *GNTI*-RNAi or *MANII*-RNAi extracts. Thus, both immunological tests of *MANII*-RNAi fruit extracts confirmed effective *core* fucose shielding by untrimmed mannoses on the α1,6 arm.

### *MANII*-RNAi Plants Do Not Show the Fruit Phenotype Characteristic for *GNTI*-RNAi

**FIGURE 5:**
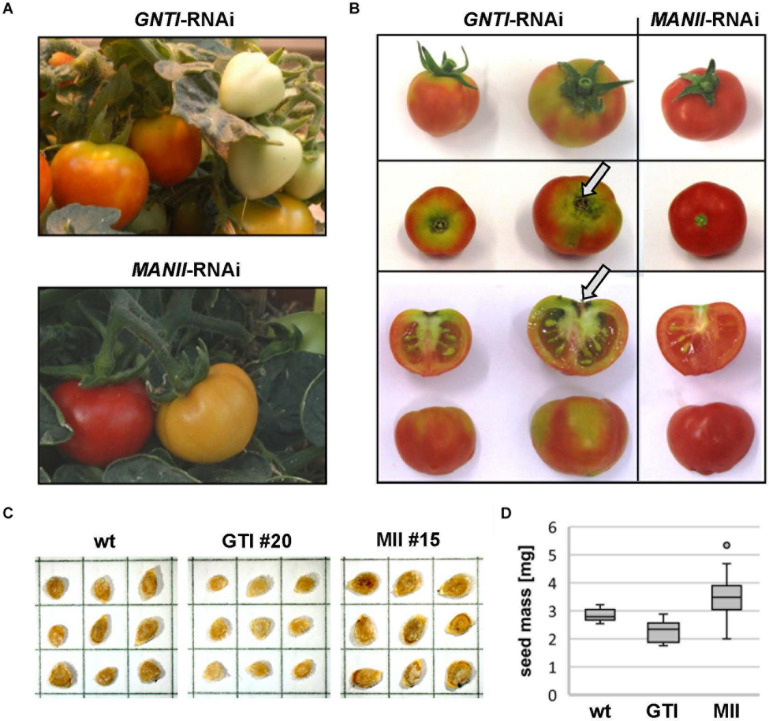
*ManII*-RNAi fruits resemble wild-type, except for seed number and mass. **(A)** Comparison of *GNTI*-RNAi (#20) and *MANII*-RNAi (#15) Micro-Tom plants in the greenhouse. Mature *GNTI*-RNAi fruits show yellow-green areas, whereas *MANII*-RNAi fruits develop normally. **(B)** Stalk-associated fruit parts of *GNTI*-RNAi (#20) remain incompletely ripe (green parts) and show signs of necrosis (arrows), whereas *MANII*-RNAi (#15) fruits look normal but contain less seeds. **(C)** Acid-washed dried and stored tomato seeds, shown for Micro-Tom wild-type (wt), and exemplarily for a *GNTI*-RNAi (GTI) and *MANII*-RNAi line (MII). Each box measures 5 mm × 5 mm. **(D)** *MANII*-RNAi seeds are heavier and *GNTI*-RNAi seeds are slightly lighter than Micro-Tom wild-type. The box plot was compiled with seeds from several seed batches (biological replicates) of wild-type (wt; *N* = 14), *GNTI*-RNAi lines (GTI #20, *N* = 8; #45, *N* = 4), and *MANII*-RNAi lines (MII #11, *N* = 13; #13, *N* = 2; #14, *N* = 23; #15, *N* = 11; #18, *N* = 9). For details (one-way ANOVA), see Supplementary Excel Sheet.

*MANII*-RNAi plants developed almost like wild-type, whereas fruits of *GNTI*-RNAi plants ripened irregularly, with a patchy coloration (from green, yellow to red) and necrotic stalk-attached parts before ripening was complete (Figures 5A,B, arrows). This is likely one reason for the early fruit drop observed when *GNTI*-RNAi plants still looked fine and green, not yet showing signs of premature senescence. By contrast, *MANII*-RNAi fruits remained attached to the plant for an extended time. In addition, larger seeds were produced (Figure 5C), however, at the expense of the seed number per fruit. Seed mass was about 1.8-fold higher compared to wild-type and *GNTI*-RNAi plants (Figure 5D). In some lines, seed number was extremely reduced, and often fruits without seeds were found (thus work was continued with lines that still produced seeds). Interestingly, for *Arabidopsis hgl1* (*manII*) and *cgl1* (*gntI*) mutants, no obvious changes in seed development were observed, with 1,000-grain weights comparable to wild-type (not shown). This pointed to a tomato-specific phenomenon perhaps due to interference with hormone crosstalk during fruit ripening. Of note, the *GNTI*-RNAi phenotypes were not overcome by back-crossing, although plants could easily be crossed with the tall wild-type variety Moneymaker (to exclude an influence of the dwarf mutant background; Supplementary Figure S6) and another Micro-Tom line (*vINV*-RNAi, not shown). An extreme *MANII*-RNAi line was used for reciprocal crosses with Micro-Tom wild-type (Supplementary Figure S7). Pollination with wild-type did not result in vital seeds, and no hygromycin-resistant progeny was found when *MANII*-RNAi was used as the pollen donor. This revealed problems for both female and male gametophytes.

Since fruits of *GNTI*-RNAi plants came loose easily, the force needed to pick a fruit was determined for the Micro-Tom genotypes at comparable ripening stages (Supplementary Figure S8). When red fruits were pulled in longitudinal direction, fruits of the wild-type and *MANII*-RNAi were released at 5.3 N, but *GNTI*-RNAi fruits already at 4.2 N. Thus, premature abscission observed for *GNTI*-RNAi plants manifested before fruits dropped naturally. Several assumptions can be made about the underlying mechanisms in the tomato *GNTI*- and *MANII*-RNAi plants, but interference with auxin versus ethylene signaling during fruit ripening would fit the observed phenotypes best.

### Ethephon Alleviates and Indole-3-Acetic Acid Mimics the Patchy Phenotype of *GNTI*-RNAi Fruits

**FIGURE 6:**
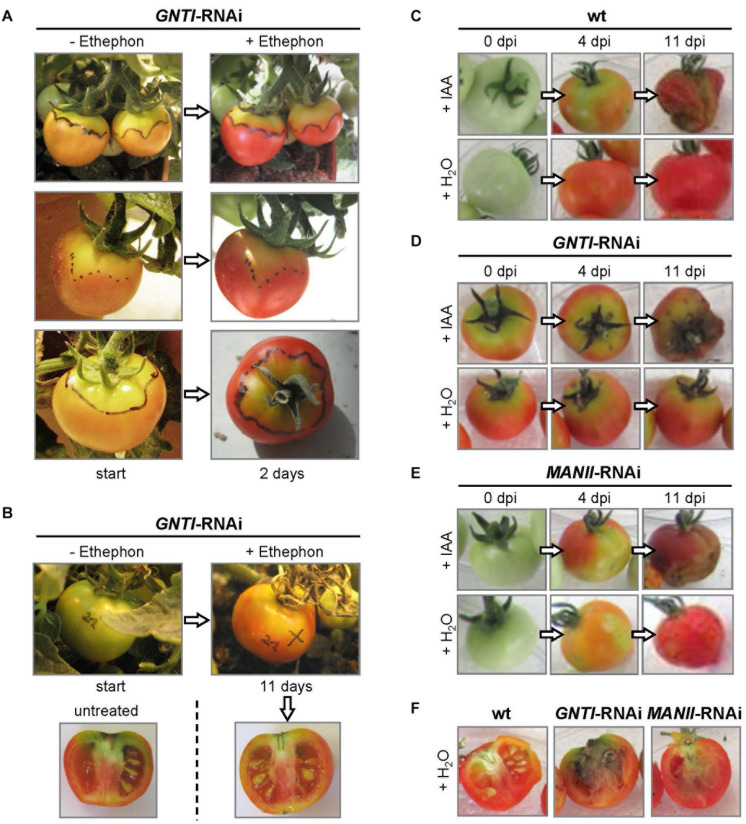
The fruit phenotype of *GNTI*-RNAi is alleviated by ethephon and mimicked by indole-3-acetic acid (IAA). **(A)** Fruits of Micro-Tom *GNTI*-RNAi line #20 were photographed before (-) and after (+) treatment with 2% ethephon (2-chloro-ethyl-phosphonic acid), whose decomposition results in ethylene release. Ethephon was applied when irregular fruit coloration appeared (the border between yellow and orange areas was marked with a water-resistant pen). After 2 days, orange areas had turned red, while yellow-green areas remained patchy yellow-green. **(B)** After ethephon treatment of green fruits, *GNTI*-RNAi fruits developed a more homogeneous red color, but necrotic stalk regions remained after 11 days, compared to an untreated fruit from the same plant. **(C–F)** Detached Micro-Tom fruits (breaker stage) were infiltrated in parallel with either tap water (+ H_2_O) or the auxin indole-3-acetic acid (+ IAA, 100 μM). Dpi, days post infiltration. After 4 days, IAA infiltration resulted in patchy coloration of both wild-type (wt) and *MANII*-RNAi fruits (#20), similar to *GNTI*-RNAi (#14) fruits. After 11 days, IAA-infiltrated fruits looked rotten. When opening the water-infiltrated fruits, signs of internal rotting were visible for *GNTI*-RNAi, and to some extent also for *MANII*-RNAi, but not for wt fruits.

Ethylene, important for fruit ripening, is also known to be involved in senescence (Lim et al., 2007) and defense (Wang et al., 2002), which appears enhanced in *GNTI*-RNAi plants (Figure 1). To address whether ethylene may influence the patchy phenotype, *GNTI*-RNAi fruits were treated with ethephon (an industrial agent used for acceleration of fruit ripening), which releases ethylene during decomposition. Ethephon was applied to fruits when separation into orange and yellow parts became visible (Figure 6A). Two days later, the orange parts had turned red, while the yellow parts remained more or less the same. When a normal-looking green fruit (of almost the size of the red fruits) was subjected to the ethephon treatment, 11 days later, the fruit showed a more homogeneous yellow-red coloration compared to an untreated fruit on the same plant, but patchiness and stalk-attached necrosis remained (Figure 6B). Hence, we reasoned that rather auxin responses may be influenced in the RNAi lines.

Many studies have shown that auxin (IAA) plays an important role in fruit ripening and aging. Exogenous IAA treatment of immature tomato fruits is known to delay ripening and results in parthenocarpy (Kumar et al., 2014). When IAA was injected into mature green fruits of Micro-Tom wild-type, fruits ripened but started to rot after 1 week, while water-infiltrated fruits of the same plant ripened without rotting (not shown). This was also observed for detached fruits. Compared to water, IAA infiltration into green wild-type fruits resulted in delayed ripening and early rotting (Figure 6C). *GNTI*-RNAi fruits kept their patchy phenotype upon IAA injection (Figure 6D), and water-infiltrated *MANII*-RNAi fruits showed a patchy coloration after 4 days, reminiscent of the *GNTI*-RNAi phenotype (Figure 6E). Internal signs of rotting were also visible for detached, water-injected fruits of *GNTI*-RNAi, and to a much lesser extent also for *MANII*-RNAi, but not for wild-type (Figure 6F).

### Evidence for Deregulated Hormone Signaling in *GNTI*-RNAi and *MANII*-RNAi Plants

**FIGURE 7:**
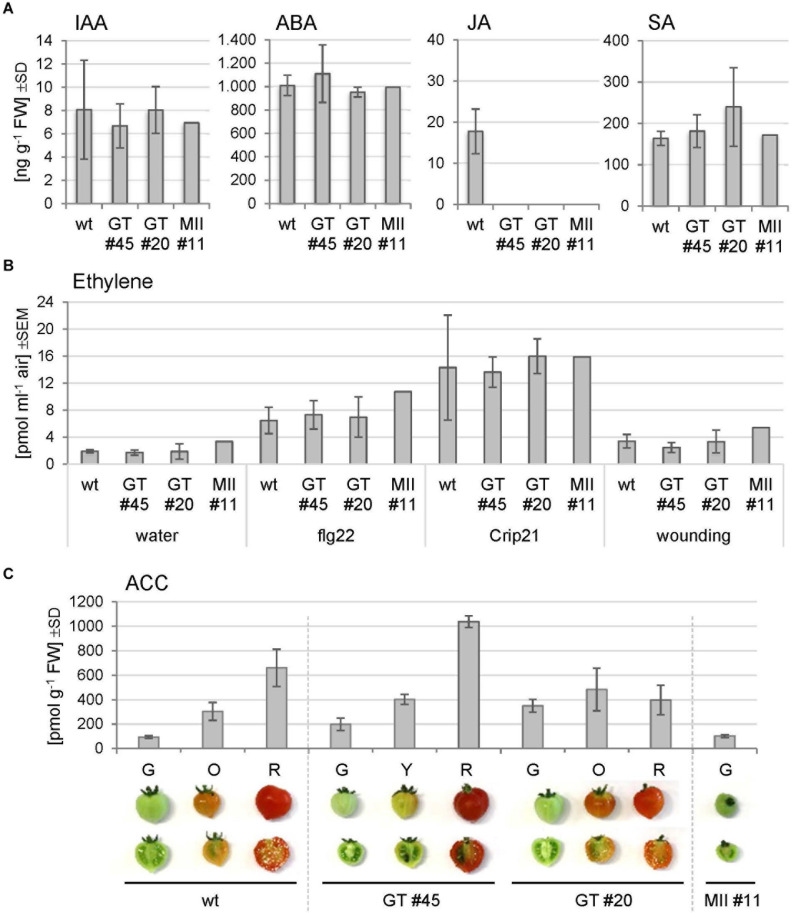
Hormone measurements of Micro-Tom leaf and fruit extracts. **(A)** Phytohormone levels were determined in leaf extracts of the wild-type (wt) and indicated RNAi lines (GT, *GNTI*-RNAi; MII, *MANII*-RNAi). IAA, indole acetic acid; ABA, abscisic acid; JA, jasmonic acid; SA, salicylic acid. Bars represent the mean of three biological replicates for three plants of wt, four of each *GNTI*-RNAi line, and two of *MANII*-RNAi line #11. SD, standard deviation. **(B)** Ethylene emission from leaf pieces after the indicated treatments: water (mock), a bacterial elicitor (1 μM flg22), a parasitic plant elicitor from Cuscuta (1 μM Crip21), or repeated squeezing with a forceps (wounding). Bars represent the mean of three measurements with three plants per genotype, i.e., Micro-Tom wild-type (wt), *GNTI*-RNAi lines (GT #20, #45), and one *MANII*-RNAi line (MII #11). SEM, standard error of the mean. **(C)** Ethylene was chemically released from 1-aminocyclopropane-1-carboxylic acid (ACC) of frozen fruit halves. Micro-Tom wild-type (wt), GNTI-RNAi lines (GT #20, #45), and MANII-RNAi line (MII #11) at the ripening stages shown (G, green; O, orange; Y, yellow; R, red). Bars represent the mean of three replicates. SD, standard deviation of the mean.

To investigate whether phytohormone levels might be altered in the RNAi lines, the contents of IAA, ABA, JA, and SA were determined in Micro-Tom leaf tissues (Figure 7A). Compared to the wild-type, no significant changes were recorded, except for JA that was not detected in the RNAi plants. SA levels were by trend slightly elevated in the *GNTI*-RNAi lines compared to wild-type and *MANII*-RNAi.

To test whether stress treatment may reveal differences between the genotypes, ethylene release was measured upon wounding or incubation with elicitors, which are perceived by the heavily N-glycosylated ectodomains of receptor-like kinases (RLKs) at the plasma membrane, i.e., bacterial flg22 by FLS2 (Häweker et al., 2010) or Crip21 by CuRe (18 potential N-glycosylation sites) of the plant pathogen *Cuscuta reflexa* (Hegenauer et al., 2016, 2020). Ethylene production was found to be highly variable in leaf samples of all genotypes, without clear differences (Figure 7B). Moreover, when chemically released from ACC of harvested fruits, the amounts correlated with the degree of fruit ripening (Figure 7C). Together, these findings pointed to a subtle interference of altered *N*-glycan modification with hormone crosstalk/signaling during fruit ripening in the RNAi lines.

**FIGURE 8:**
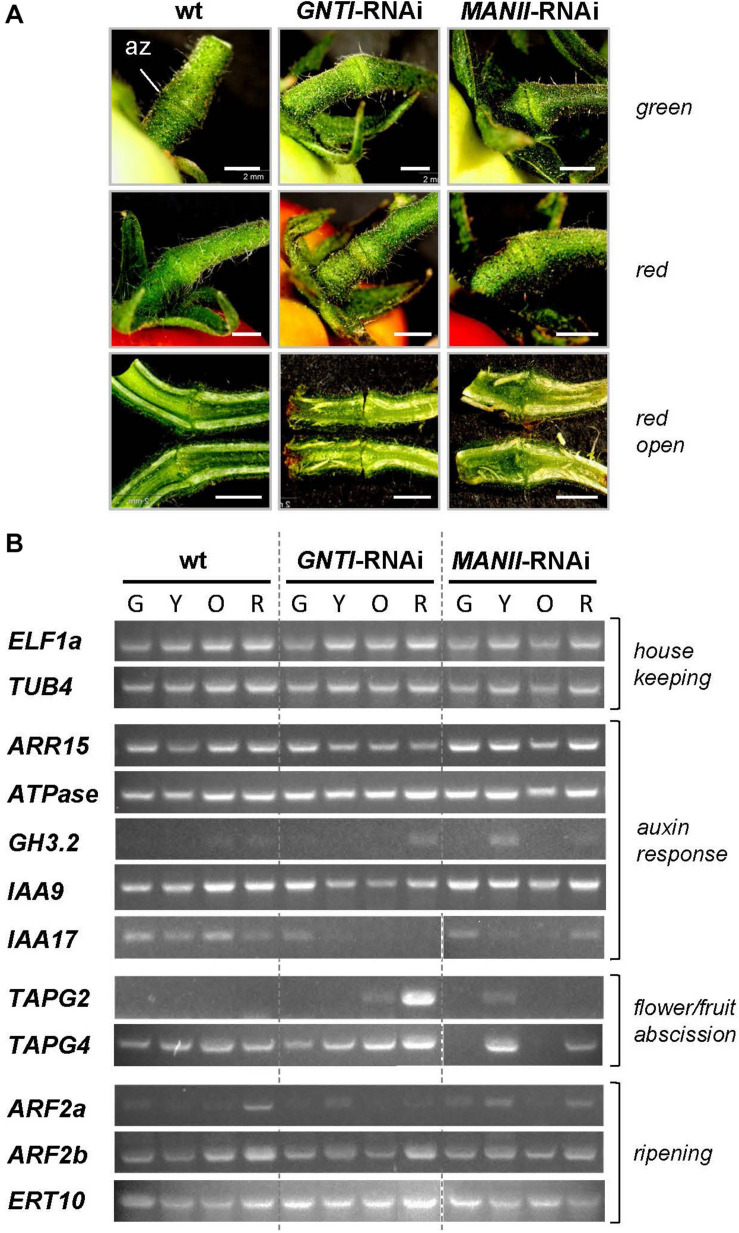
Marker gene expression in wild-type, *GNTI*-RNAi, and *MANII*-RNAi fruit pedicels. **(A)** Close-up view of pedicels attached to green (top) or red (ripe) fruits of Micro-Tom wild-type (wt), *GNTI-*RNAi (#20), and *MANII*-RNAi (#18). Parts ca. 3 mm to both sides of the abscission zone (az) were used for RNA isolation. Longitudinally cut open pedicels of red fruits are shown below. Fruits were attached to the left. Bars = 2 mm. **(B)** Semiquantitative RT-PCR of abscission zones of *GNTI*-RNAi (#20) and *MANII*-RNAi (mix of #11 and #18) with green (G), yellow (Y), orange (O), or red (R) fruits was performed with two housekeeping genes as reference: *ELF1a*, translation elongation factor; *TUB4*, tubulin 4 (top). Marker genes were chosen based on known responses to auxin/ethylene and expression during certain developmental stages (abscission, fruit ripening). *ARR15*, *Arabidopsis* response regulator type A, auxin-responsive-negatively regulating cytokinin; *ATPase* (H^+^), auxin/sugar-responsive; *GH3.2*, IAA-amido synthetase, auxin- and ethylene-responsive; *IAA9/17*, AUX/IAA transcription factors: *IAA9*, auxin-responsive, ethylene-repressed; important for leaf morphology/fruit set; *IAA17*, auxin-responsive, ethylene-repressed; *TAPG2/4*, tomato abscission-related polygalacturonases; *ARF2a/b*, auxin response factors important for tomato fruit ripening (auxin versus ethylene); *ERT10*, tomato ripening-related marker.

To shed more light on altered hormone responses, we chose pedicels as investigation object. With respect to the observed early fruit drop in *GNTI*-RNAi lines, tissue around the az of green (G), yellow (Y), orange (O), and red (R) fruits (Figure 8 and Supplementary Figure S3) was used for semiquantitative RT-PCR analyses. Harvested parts are shown for green and mature red fruit-bearing plants of wild-type, *GNTI*-RNAi, and *MANII*-RNAi lines without visible changes in the pedicel (Figure 8A) but obvious differences in the attached fruits. Those of the *GNTI*-RNAi line exhibited the patchy ripening phenotype linked with necrotic signs in the region where the stalk was attached and in partly brown regions around the seeds. The *MANII*-RNAi line developed completely red fruits (Supplementary Figure S3). Semiquantitative RT-PCR analyses were conducted for several target genes with focus on auxin-responsiveness. Marker genes for either ripening or abscission were also included. Compared to two housekeeping genes (*ELF1a* and *TUB4*), known auxin-response and az-marker genes were found to be deregulated in the RNAi lines. Tomato *ARR15* (*Arabidopsis thaliana* response regulator type A), induced by auxin-negatively regulating cytokinin (Leibfried et al., 2005; To et al., 2007; Müller and Sheen, 2008) accumulates during fruit ripening in the az of wild-type, declined in the *GNTI*-RNAi lines and remained at elevated levels in *MANII*-RNAi lines. H^+^ *ATPase* (LHA4; Mito et al., 1996) was more prominently expressed in the early ripening stages of both RNAi lines, whereas *GH3.2* (group II IAA-amido synthetase) that is auxin- and ethylene-responsive (Sravankumar et al., 2018) showed a peak in the az of yellow *MANII*-RNAi fruits and seemed to be more present in red fruits of *GNTI*-RNAi lines compared to those of wild-type. AUX/IAA repressor *IAA9*, shown to decrease in az upon flower removal (Meir et al., 2010), decreased in *GNTI*-RNAi (stages Y and O) but showed high expression in *MANII*-RNAi, similarly to wild-type. *IAA9* and, to a higher extent, *IAA17* are both induced by auxin and repressed by ethylene (Audran-Delalande et al., 2012). In wild-type, *IAA17* was expressed during all ripening stages, but only detected in the az of green fruits for *GNTI*-RNAi, and in those of both green and red fruits of *MANII*-RNAi plants. Az markers *TAPG2* and *TAPG4* (two tomato abscission-related polygalacturonases; Meir et al., 2010; Reichardt et al., 2020) were clearly elevated in *GNTI*-RNAi plants (especially *TAPG2* in the az of red fruits), with deregulation in *MANII*-RNAi (high in the az of yellow fruit pedicels). Auxin-response factors (ARFs) ARF2a (ethylene-responsive) and ARF2b (auxin-responsive) are important during Micro-Tom fruit ripening (Hao et al., 2015). Differential expression in the RNAi lines was detected for *ARF2a* with highest levels in red fruit pedicels of the wild-type, yellow ones in *GNTI*-RNAi, and a mixture of both in *MANII*-RNAi. Ripening-related marker *ERT10* (Meir et al., 2010; Reichardt et al., 2020) was elevated in the az of *GNTI*-RNAi and deregulated in those of *MANII*-RNAi plants (inverse to wild-type in the az of green fruit pedicels).

### Glycoprotein Turnover in *N. benthamiana* Wild-Type Versus *GNTI*-RNAi and *MANII*-RNAi Plants

To address the possibility that secreted glycoproteins with altered *N*-glycan structures may be less stable in the RNAi plants, *N. benthamiana* (suitable for agroinfiltration) was stably transformed with *GNTI*-RNAi or *MANII*-RNAi constructs (Supplementary Figure S1A). Reduced cgly patterns were confirmed by immunoblotting in the T1 and T2 generation (not shown). To prevent KOR1 cycling between the plasma membrane and *trans*-Golgi network (Nagashima et al., 2020), a binary KOR1-GFP construct with masked C-terminus was cloned (Supplementary Figure S9A) and detected at the cell surface of transfected protoplasts (Supplementary Figure S9B), similar to GFP-KOR1 (von Schaewen et al., 2015). Immunoblot analysis of agroinfiltrated *N. benthamiana* wild-type leaves (Supplementary Figure S9C) showed similar expression for KOR1-GFP next to GFP-KOR1 (Rips et al., 2014).

**FIGURE 9:**
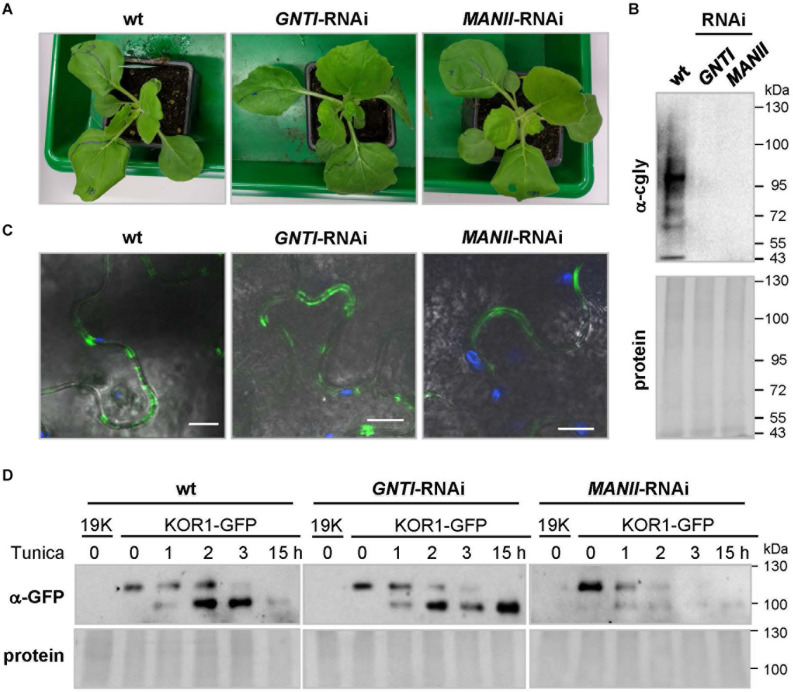
Analysis of glycoprotein stability in *N. benthamiana GNTI*- and *MANII*-RNAi plants. **(A)** *N. benthamiana* wild-type (wt), *GNTI*-RNAi (#10), and *MANII*-RNAi (#4) T3 plants prior to agroinfiltration. **(B)** Immunoblot analysis of indicated leaf extracts (membrane fractions) developed with anti-complex glycan antiserum (α-cgly = α-PHA-L). The Ponceau S-stained blot (protein) is shown as the loading reference. Apparent molecular masses are indicated in kDa (PageRuler Prestained Protein Ladder, Fermentas). **(C)** Confocal laser scanning microscopy (CLSM) images of KOR1-GFP expression in *N. benthamiana* wild-type and the indicated RNAi lines. Merging of GFP (green) and chlorophyll autofluorescence (blue) with bright field. Bars = 10 μM. **(D)** Immunoblot analysis of leaf extracts (membrane fractions) upon agroinfiltration and development with anti-GFP antiserum (α-GFP). Tunicamycin (Tunica, 10 μM) treatment results in the accumulation of non-glycosylated KOR1-GFP (band shift). The fading top bands indicate decay due to protein turnover. 19K, co-expressed *Agrobacterium* silencing suppressor strain (negative control). The Ponceau S-stained blots are shown as the protein loading reference. Molecular masses are indicated in kDa (PageRuler Prestained Protein Ladder, Fermentas).

*Nicotiana benthamiana* wild-type plants in the greenhouse, cultivated together with *GNTI*- and *MANII*-RNAi lines (T3, Figure 9A), showed reduced cgly patterns (Figure 9B). KOR1-GFP was agroinfiltrated into leaves of the wild-type, *GNTI*-RNAi, and *MANII*-RNAi plants. When KOR1-GFP signals were detected at the plasma membrane of all genotypes (Figure 9C), leaves were infiltrated with water (mock) or tunicamycin (10 μM, to abrogate *N*-glycosylation and prevent further secretion) and harvested over the following hours. Immunoblot analyses with anti-GFP antiserum (α-GFP) indicated that KOR1 may be slightly less stable in the RNAi plants compared to wild-type (Figure 9D). This was deduced from persistence of the top bands versus the newly emerging bottom bands over time and most evident 1–2 h after tunicamycin infiltration.

## Discussion

Here, we compared RNAi suppression of two consecutive glycosyltransferases in the Golgi apparatus of tomato that either prevent (*GNTI*-RNAi) or alter (*MANII*-RNAi) complex *N*-glycan formation on secretory glycoproteins. Compared to *GNTI*-RNAi (resulting in signs of premature senescence, early fruit drop, and patchy fruit ripening), *MANII*-RNAi silencing was far better tolerated in tomato, which is likely due to the hybrid nature of the accumulating *N*-glycans. Nevertheless, *MANII*-RNAi fruit extracts showed reduced recognition by cgly-specific antibodies (similar to *GNTI*-RNAi), which independently confirmed shielding of *core* fucoses (by branched mannoses on the 1,6-arm) and an altered position of the xylose residue, as described for the corresponding *Arabidopsis* mutants (Kaulfürst-Soboll et al., 2011b). Subtle phenotypic differences were also observed for the Micro-Tom *MANII*-RNAi plants: first of all, more vigorous growth, visible as thicker stems and broader leaves—sometimes with less complex shape that rolled up their rims during the ripening stage (Figure 1 and Supplementary Figure S3). The latter is reminiscent of tomato *dr12* lines with defective ARF, whose mRNA accumulation is ethylene-dependent (highest in early red fruits) but also important for seed development and seedling growth. Mutant *dr12* plants showed upwardly curled leaves (Jones et al., 2002). Furthermore, the pectin fine structure and tissue architecture were altered (Guillon et al., 2008), which may also be affected in the RNAi plants. Interestingly, a blotchy fruit phenotype was also observed for isopentenyltransferase *IPT*-transformed tomato lines with enhanced cytokinin levels (Martineau et al., 1994; reviewed in Srivastava and Handa, 2005), which may hint at a link to cytokinin, as reported for rice *gntI* mutants (Fanata et al., 2013). Of note, severe ripening defects with yellow and orange patches (never reaching the typical red color of wild-type fruits, similar to *GNTI*-RNAi) were also observed for Micro-Tom *ARF2ab*-RNAi lines that are compromised in normal climacteric fruit ripening (auxin versus ethylene; Hao et al., 2015). Clearly, flowers of *MANII*-RNAi plants were bigger and produced fleshier fruits (Supplementary Figure S3) with matte appearance that showed earlier water loss than detached wild-type fruits (Figure 1). Here, one could speculate that the latter reflects altered cuticular wax precursor synthesis/transport that involves the ER and Golgi (reviewed in Trivedi et al., 2019). Moreover, *MANII*-RNAi fruits contained fewer, but larger, seeds. As observed during back-crossing, both male and female fertility was compromised, resulting in fruits with less or no seeds in several independent lines. Seedlessness is an attribute of parthenocarpy that develops when male or female gametophytic plant fertility is impaired. This is often accompanied by an untimely increase of auxins and gibberellins in the ovaries (Nitsch, 1970; George et al., 1984; Mazzucato et al., 1998), e.g., in tomato *pat* mutants that show accelerated ripening and higher fruit quality, but produce no seeds due to aberrant anther and ovule development (Mazzucato et al., 1998). Of note, parthenocarpy can be induced by application of auxin and gibberellin (Pandolfini, 2009) and was achieved in Micro-Tom by ectopic expression of genes for auxin synthesis or auxin responsiveness (Martinelli et al., 2009). Moreover, when red fruits of the tomato variety Ailsa Craig were *Agrobacterium*-infiltrated with the *GNTI*-RNAi construct, fruits dropped within a few days, but those infiltrated with the *MANII*-RNAi construct remained on the plant until harvested after 1–2 weeks (not shown). Thus, the observed phenotypes seem not to be cultivar-specific nor the result of a specific integration site within the genome.

The largely opposing fruit phenotypes of *GNTI*- and *MANII*-RNAi tomato plants suggest that auxin versus ethylene signaling/readout, most important during the last fruit ripening stages in tomato (Srivastava and Handa, 2005), might be affected. Indeed, fruit treatments using either ethephon or IAA point in this direction, whereas the measurement of hormone contents in leaves of Micro-Tom wild-type versus the RNAi lines showed only minor differences—except for JA that was absent from the RNAi lines. The latter may be a side effect of the slightly elevated SA levels detected in the *GNTI*-RNAi lines.

Failure to provoke differential ethylene release from leaves by various stress treatments further corroborated a specific role for complex-type *N*-glycans during tomato fruit ripening. In this context, it is interesting that tomato ACC-synthase2 tilling mutants, either overproducing (*acs2-1*) or underproducing (*acs2-2*) ethylene, showed opposite phenotypes (Sharma et al., 2020). Similar to the *GNTI*-RNAi lines, *acs2-1* overproducers displayed faster leaf senescence (besides accelerated fruit ripening), but *acs2-2* underproducers slower leaf senescence (besides prolonged fruit ripening), partially reminiscent of the *MANII*-RNAi lines. Since fruit ACC contents correlated with the ripening state in all genotypes, the underlying mechanism likely acts upstream.

Subtle differences of hormone crosstalk in the Micro-Tom RNAi lines became evident when pedicel tissue around the az was used for semiquantitative RT-PCR analyses. Auxin-responsive genes were differentially regulated in *GNTI*-RNAi and *MANII*-RNAi versus wild-type. Especially the decline of tomato *ARR15* (an auxin-response regulator that negatively regulates cytokinin responses) in *GNTI*-RNAi, and its elevated expression in *MANII*-RNAi, correlates with the observed early fruit drop in *GNTI*-RNAi, which suggests a less or higher suppression of cytokinin-responsive genes, respectively. The low abundance of *IAA9* and *IAA17* mRNA expression (auxin-induced, ethylene-repressed) in the *GNTI*-RNAi lines is indicative of elevated ethylene levels, likely induced by too much auxin/signaling (Abeles and Rubinstein, 1964). In *MANII*-RNAi, az of the orange ripening stage showed low amounts of *IAA17* transcript compared to wild-type, but similar levels in the red stage, hinting at less ethylene in ripe fruits of *MANII*-RNAi compared to *GNTI*-RNAi. This is in accordance with the higher transcript levels of the auxin-conjugating enzyme GH3.2 in the red fruit stage of *GNTI*-RNAi, whereas in *MANII*-RNAi, only a temporary increase was observed for the yellow stage.

Compared to wild-type, abscission markers *TAPG2* and *TAPG4* were both elevated in *GNTI*-RNAi (especially TAPG2) but seemed deregulated in *MANII*-RNAi. Moreover, ARF2a and ARF2b act as repressors of auxin-responsive genes in tomato, but only ARF2a is induced by ethylene (Hao et al., 2015). Interestingly, the az of both *GNTI*- and *MANII*-RNAi pedicels showed elevated ARF2a levels during the early ripening stages, hinting at elevated ethylene/readout. However, ripening marker *ERT10* was much higher expressed in *GNTI*-RNAi and inversely regulated in *MANII*-RNAi (highest in the az of green fruits, i.e., opposite to wild-type). Thus, alterations in auxin signaling (as indicated by the DII Venus sensor in the roots of *Arabidopsis* cgly mutant seedlings; Frank et al., 2021) would best explain the observed phenotypic deviations, which should be linked to the different *N*-glycan structures.

Early investigations by Handa et al. (1985) deduced a possible effect of *N*-glycans on hormone crosstalk from experiments, in which abrogation of *N*-glycosylation by tunicamycin treatment interfered with fruit ripening. Later, application of free Man5GlcNAc was shown to prevent the inhibitory effect of tunicamycin, suggesting an involvement of released *N*-glycans (Yunovitz and Gross, 1994b). Indeed, experiments with unbound *N*-glycans had concentration-dependent stimulatory or inhibitory effects on tomato fruit ripening (Yunovitz and Gross, 1994a, b; Yunovitz et al., 1996). Of note, M3XF [Man3(Xyl) GlcNAc (Fuc)GlcNAc], characteristic for complex *N*-glycans in wild-type (regardless of the presence or absence of terminal GlcNAc residues), and Man5GlcNAc (M5), representing the *N*-glycan structure of *GNTI*-RNAi plants, were equally able to promote fruit ripening and senescence at elevated concentrations. Especially, M5 was found to have an inhibitory effect (Napier and Venis, 1991; Yunovitz and Gross, 1994a). Based on these criteria, *N*-glycans of the studied RNAi lines differ only by the absence (*GNTI*-RNAi) or presence (*MANII*-RNAi) of *core* fucose and xylose residues.

**FIGURE 10:**
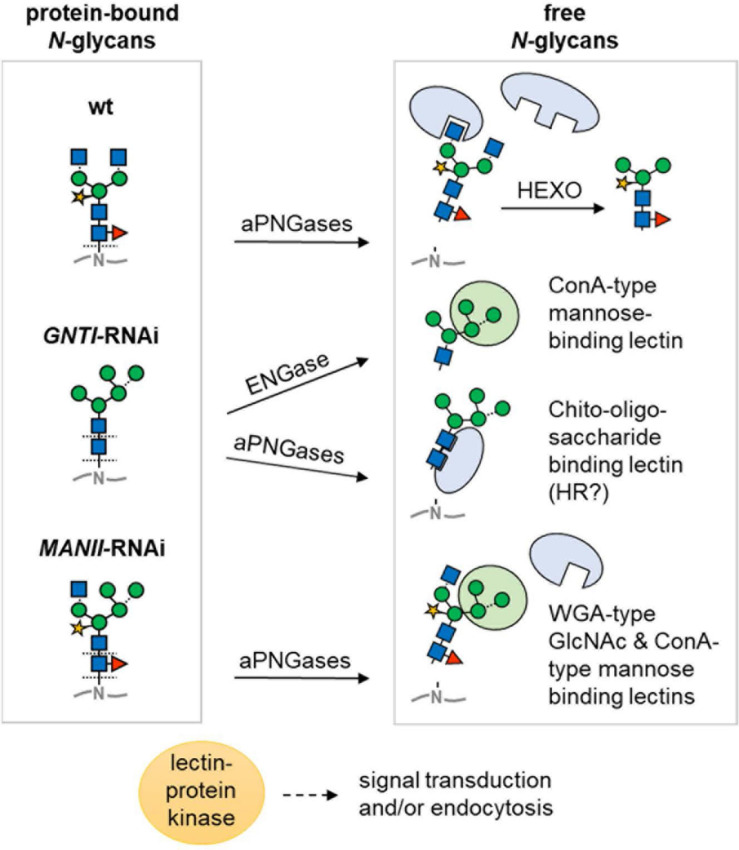
Model of differential *N*-glycan interpretation in the RNAi lines. Protein-bound *N*-glycans of wild-type (wt), *GNTI*-RNAi, or *MANII*-RNAi plants (left) may be either directly bound or liberated depending on capability for enzymatic release. Decoding is suspected to occur by proteins with lectin domains (for sugar symbols, see Figure 3A). Arrows indicate cleavage by peptide:N-glycosidase (PNGase A isoforms in different acidic compartments that may release *core* fucosylated N-glycans) or in the cytosol by endo-β-*N*-acetyl-glucosaminidase (ENGase). In *GNTI*-RNAi plants, the chito-oligosaccharide N-glycan *core* may be decoded upon release from the glycoprotein backbone, potentially resulting in HR (hypersensitive response). Abbreviations: ConA, concanavalin A; HEXO, hexosaminidases (apoplast/vacuole); WGA, wheat-germ agglutinin.

Another aspect is the ability of plant cells to enzymatically release sugar moieties or entire *N*-glycans in post-Golgi acidic compartments. ENGase and peptide:N-glycanase (PNG1 in *Arabidopsis*; Diepold et al., 2007) are confined to the cytosol, where they aid in the clearance of misfolded glycoproteins upon retro-translocation from the ER. To date, it is not clear whether and how these activities might also contribute to tomato fruit ripening (Nakamura et al., 2008; Maeda and Kimura, 2014). However, two aPNGase isoforms (Uemura et al., 2018) that are similar to PNGase A from almond, which can release *core* fucosylated *N*-glycans (Hossain et al., 2010), are probably located in acidic compartments of tomato fruits (apoplast/vacuole). Thus, they may release both complex-type (with *core* fucose) and high mannose-type *N*-glycans from secreted glycoproteins (Figure 10). We speculate that those released in wild-type and *MANII*-RNAi plants would carry *core* fucoses, whereas those of *GNTI*-RNAi plants may look “dangerous,” due to their accessible GlcNAc2 chitobiose part, which resembles the pathogen-associated molecular pattern PAMP chitin.

Prior to cleavage by hexosaminidases (HEXO), present in the apoplast and vacuole, wild-type *N*-glycans carry two terminal GlcNAc residues (with or without Lewis-a epitopes). Those of *MANII*-RNAi plants may only display one (on the 1,3-arm); *GNTI*-RNAi plants, none. This, and the presence of terminal mannoses on the 1,6-arm, is probably decoded by lectins (or proteins with lectin domains) that bind to terminal GlcNAc (WGA-type), mannose (ConA-type), or chitobiose units (GlcNAc2 without *core* fucose). Hence, immediately upon secretion, glycoproteins of the wild-type look “young”; those of *MANII*-RNAi, “intermediate”; and those of *GNTI*-RNAi plants, “old” (as if HEXO already clipped the terminal GlcNAc residues). This might determine their clearance rate from the plasma membrane/apoplast by endocytosis and explain why KOR1-GFP turnover was slightly accelerated in the *N. benthamiana* RNAi plants. Besides, also presence (in wild-type), absence (in *GNTI*-RNAi), or shielding of *core* fucoses (in *MANII*-RNAi) may alter signaling from the plasma membrane. One example is the transforming growth factor (TGF)-beta receptor of mammalian cells for which lack of *core* fucose caused aberrant lung development in mice (Wang H. et al., 2005; Wang X. et al., 2005). Possibly this is also the case in plants; however, not much is known about the role of *core* fucose yet in this emerging field of research (reviewed in Bellande et al., 2017; Van Holle and Van Damme, 2018).

Apparently, phytohormone signaling can be influenced by released *N*-glycans (for review, see Maeda and Kimura, 2014; Lannoo and van Damme, 2015). Since free *N*-glycans were also found in the xylem sap of tomato stems (Faugeron et al., 1999; Tsujimori et al., 2019), even systemic signaling throughout the entire plant is conceivable. We believe that a different capacity to release *N*-glycans in the *GNTI*-RNAi versus the *MANII*-RNAi lines could be linked to their opposite phenotypes in tomato. Accessibility of the chitobiose part in the *GNTI*-RNAi lines may additionally modulate growth and developmental readouts, especially concerning apoplastic lectins or receptor-like kinases with a lectin domain (LecRLKs; Bellande et al., 2017; Sun et al., 2020). In case of the terminal *N*-glycan parts, lectins with GlcNAc- or mannose-specific domains (WGA- or ConA-type, respectively; Figure 10) may compete for binding in *MANII*-RNAi plants—due to the hybrid *N*-glycan structure with one branched mannose arm—shared with *GNTI*-RNAi, and thus differ from wild-type.

Compared to wild-type, the phenotypes observed in *MANII*-RNAi Micro-Tom plants, showing increased ectopic growth and delayed senescence, may reflect stimulatory auxin-like effects, while in *GNTI*-RNAi plants, auxin-like signaling seems to be supra-optimal (“over the top”). Too much auxin, and maybe also auxin-like effects, influences ethylene signaling, as deduced from our RT-PCR results. Together with additional defense signaling in the *GNTI*-RNAi lines, this may be responsible for the observed cell death/necrosis. The latter is known to be spontaneously triggered in plant autoimmune responses that may be elicited *via* chitin elicitor receptor kinase CERK1 after pathogen attack (reviewed in van Wersch et al., 2016; Chakraborty et al., 2018). But also independent of a chitin-signaling cascade, other possibilities of chitobiose perception exist in the plant apoplast (Vanholme et al., 2014).

Concerning performance in the field, independent of the hormonal aspects, higher susceptibility of *GNTI*-RNAi plants to bacterial infection (observed in the greenhouse; for a recent review on glycan-based plant-microbe interactions see Wanke et al., 2021) may relate to the fact that bacterial chitinases can liberate *N*-glycans for nutritional purposes (Frederiksen et al., 2013). Here, it remains to be investigated whether lack of *core* fucose in *GNTI*-RNAi plants may render them an “easier” food source.

## Conclusion and Outlook

Several attempts in the field of glyco-engineering have been made concerning the reduction of unwanted side effects of plant-derived glycoproteins. Although promising, not all seem to be compatible with normal plant growth and development. In this and earlier work (Kaulfürst-Soboll et al., 2011a), we show that not all findings obtained with *Arabidopsis* (a genetic model organism) can be transferred to crops. For example, salt-stress assays of the tomato RNAi lines did not reflect the results previously obtained with the corresponding *Arabidopsis* cgly mutants. To the contrary, *MANII*-RNAi seedlings showed longer root growth on salt perhaps due to their enlarged seeds (with more storage reserves), while *GNTI*-RNAi seedlings performed equally to wild-type. The phenotypes observed in tomato during fleshy fruit development stress the relevance of complex *N*-glycan structures for/during hormonal crosstalk, most likely interfering with auxin, also in Arabidopsis roots (Frank et al., 2021). In light of our results, the previously reported cytokinin hyposensitivity of the rice *gntI* T-DNA mutant (Fanata et al., 2013) may also be caused by auxin-like effects. This matches the more recent finding that *core* fucose is important for basipetal auxin transport and gravitropic responses in rice (Harmoko et al., 2016). For clearer results, clustered regularly interspaced short palindromic repeats/caspase 9 (CRISPR/Cas9) lines should be generated and studied. Whether tomato fruits of *GNTI*-CRISPR plants will ripen at all, and *MANII*-CRISPR plants will still be able to reproduce, remains to be shown.

## Data Availability Statement

The original contributions presented in the study are included in the article/Supplementary Material. Further inquiries can be directed to the corresponding author/s.

## Author Contributions

AvS and HK-S designed the study and wrote the manuscript. HK-S, MM-B, MA, and AvS performed the experimental work. HK-S, MM-B, RB, MA, and AvS analyzed the data. All authors approved the manuscript.

## Conflict of Interest

The authors declare that the research was conducted in the absence of any commercial or financial relationships that could be construed as a potential conflict of interest.
