# Supplementary material for: Complex N-Glycans Are Important for Normal Fruit Ripening and Seed Development in Tomato
Source: Front Plant Sci. 2021 Mar 9;12:635962. doi: 10.3389/fpls.2021.635962 (PMC7985349; doi:10.3389/fpls.2021.635962)
Supplement: Supplementary file 1 [file Table_1.docx]

**Supplemental data**

**Supplemental Tables**

**Table S1. Oligonucleotide primers used in this study (restriction sites underlined).**

| **Name** | **Sequence** | **Construct/**  **Application** |
| --- | --- | --- |
| ***ManII*-RNAi** | | |
| ManII-RNAi s_SalI | CACCGTCGACAGTCCAAGCACATCCTAGATA | sense-intron-antisense |
| ManII-RNAi as_BamHI | NNNGGATCCAAATTCTGGTTTAAAGCCA |  |
| ManII-RNAi s_BamHI | CACCGGATCCAAGCACATCCTAGATATGTTG | antisense-intron-sense |
| ManII-RNAi as_SalI | NNNGTCGACCAAATTCTGGTTTAAAGCCA |  |
| ***GNTI-*RNAi for *S. lycopersicon*var. Moneymaker Micro-Tom and *N. benthamiana*** | | |
| GNTI-RNAi s-SalI | N6-GTCGACCAATTAAGGGCTCTTGTTC | Sense-intron-amtisense |
| GNTI-RNAi as-BamHI | N6-GGATCCGGCCACTTTGGAG |  |
| **Semi-quantitative RT-PCR** | | |
| elf1a-1272F  EF-1a (Solyc06g009960/70) | AGCCCATGGTTGTTGAGACCTTTG | Provided by Andreas Schaller (University of Hohenheim, Germany)  32 cycles |
| elf1a-1461R  EF-1a(Solyc06g009960/70) | TTCGAAACACCAGCATCACACTGC |  |
| tub4-317F  b-tub (Solyc04g081490) | TAGAGCCTGGTACGATGGATAG | Provided by Andreas Schaller (University of Hohenheim, Germany)  32 cycles |
| tub4-450R  b-tub (Solyc04g081490) | CAACTCAGCGCCTTCAGTATAA |  |
| TAPG2-385F(Solyc02g067640) | TGGGCTTGCAAGAACTCCAA | Provided by Andreas Schaller (University of Hohenheim, Germany)  38 cycles |
| TAPG2-574R(Solyc02g067640) | TGGGACTATTTCCTGGAGCG |  |
| TAPG4-151F(Solyc12g096750) | TCTACGAGCGCCTCCACTA | Provided by Andreas Schaller (University of Hohenheim, Germany)  38 cycles |
| TAPG4-390R(Solyc12g096750) | GCAAGCCCAAAGACTAGCAC |  |
| ERT10F  X72730 | TGCATATGCGCGCTGCTAATCAAG | Provided by Andreas Schaller (University of Hohenheim, Germany)  38 cycles |
| ERT10R  X72730 | GAGGGCCAACACCTTTGTAATGCT |  |
| Sola-GH3-273bp_F | tcgtcgccagctactttaca | 36 cycles |
| Sola-GH3-273bp_R | agaccgcagagcatttgagt |  |
| Sl.ATPase-138bp_F | gtttcttcacggacaaatttgg | 30 cycles |
| Sl.ATPase-138bp_R | caacgaaggaccagcttcg |  |
| SlIAA9-F (Solyc04g076850.2.1) | TGGCCACCCATTCGATCTTTTAG | 36 cycles |
| SlIAA9-R Solyc04g076850.2.1 | CGCAACACACATTAGTTTGCAG | 36 cycles |
| SlIAA17-320bp_F | taatgttaatggtaaaagagg | 35 cycles |
| SlIAA17-320bp_R | ctttcattccttggacaata |  |
| StARR-15(227bp)_F  (Soly03g113720.3.1) | cctcgcattgatagatgtttg | LOC101260711  34 cycles |
| StARR-15(227bp)_R  (Soly03g113720.3.1) | tcaatgtcatgggctgtaact |  |
| SlARF2a_F  Solyc03g118290.2.1 | GCCTAGATCCAGTATTTTGCC | 36 cycles |
| SlARF2a_R | GAGGAGCCAAAACCTGATAAC |  |
| SlARF2b_F Solyc12g042070.1.1 | CGATCAAATGTTCTGCCCTCG | 36 cycles |
| SlARF2b_R | ACATCAGCCTTCTCGTCATC |  |
