# Supplementary material for: Complex N-Glycans Are Important for Normal Fruit Ripening and Seed Development in Tomato
Source: Front Plant Sci. 2021 Mar 9;12:635962. doi: 10.3389/fpls.2021.635962 (PMC7985349; doi:10.3389/fpls.2021.635962)
Supplement: Supplementary file 2 [file Data_Sheet_1.pdf]

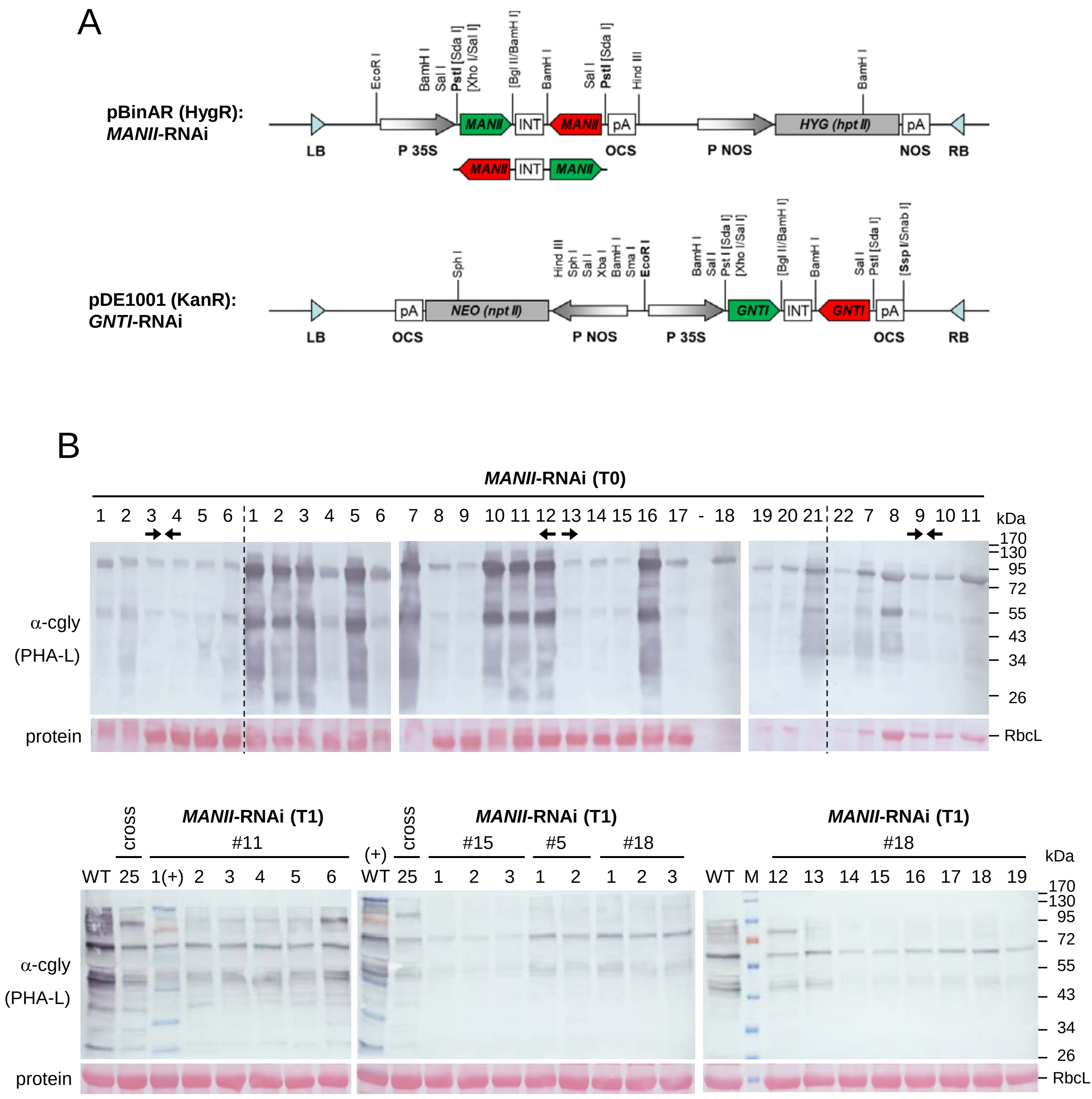

cross: *GNTI*-RNAi x *vINV*-RNAi
(+) : with Marker (M)
RbcL: Rubisco Large subunit

C

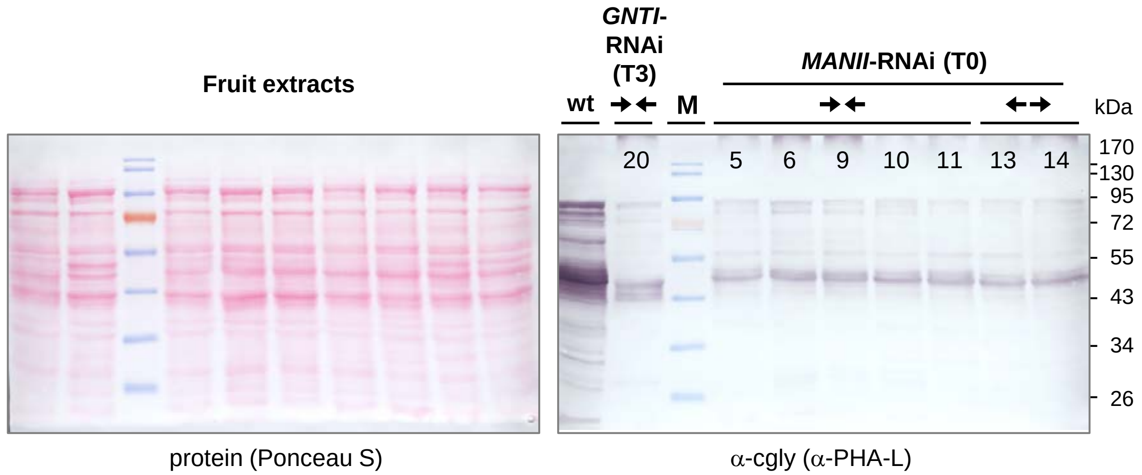

Figure S1. Binary expression cassettes and analysis of Micro-Tom *MANII*-RNAi transformants.

A, *MANII*-RNAi constructs with opposite orientation of the sense and antisense fragments relative to the intron (INT) were assembled in pUC-RNAi. The PstI fragment (bold) was inserted into SdaI-opened binary vector pBinAR (HygR) and used for Agrobacterium-mediated transformation of Moneymaker Micro-Tom. The sense-intron-antisense construct was also used for MANII suppression in *N. benthamiana*. For GNTI, a similar region previously used for tomato *GNTI*-RNAi (Kaulfürst-Soboll et al. 2011a) was amplified from *Nicotiana tabacum* SNN clone A9 (Wenderoth and von Schaewen 2000) and assembled in pUC-RNAi. The EcoRI/SspI fragment (bold) was inserted into EcoRI/SnaBI-opened binary vector pDE1001 (KanR) and used for Agrobacterium-mediated *N. benthamiana* transformation. LB/RB, left/right T-DNA border. Hpt/npt, hygromycin (HYG)/neomycin (NEO) phosphotransferase; OCS/NOS, Octopine/Nopaline synthase; P, promoter; 35S, constitutive promoter of Cauliflower Mosaic Virus (CaMV); pA polyadenylation signal.

B, Immunoblot analysis (colorimetric development) of leaf extracts (primary transformants, T0; progeny of selected lines, T1), expressing the indicated RNAi constructs. Cross: *GNTI*-RNAi x *vINV*-RNAi; (+) with Marker (M); RbcL: Large subunit of Ribulose-bisphosphate carboxylase/oxygenase (Rubisco).

C, Immunoblot analysis of fruit extracts from selected primary transformants (T0), expressing the indicated RNAi constructs.

B-C, Blotted extracts were developed colorimetrically (as described in Wenderotth and von Schaewen, 2000) with a complex glycan antiserum (α-cgly) raised against bean lectin PHA-L (mainly binding to β1,2-xylose but also *core* β1,3-fucose residues) in rabbits. Compared to wild-type (wt), both *MANII*-RNAi constructs (A, sense = green, antisense = red fragment orientations, indicated by arrows) led to similar reduction of cgly recognition as in *GNTI*-RNAi (T3). The Ponceau S-stained blots (protein) are shown as loading reference. Apparent molecular masses are indicated in kDa (PageRuler Prestained Protein Ladder, Fermentas).

Supports Figure 1

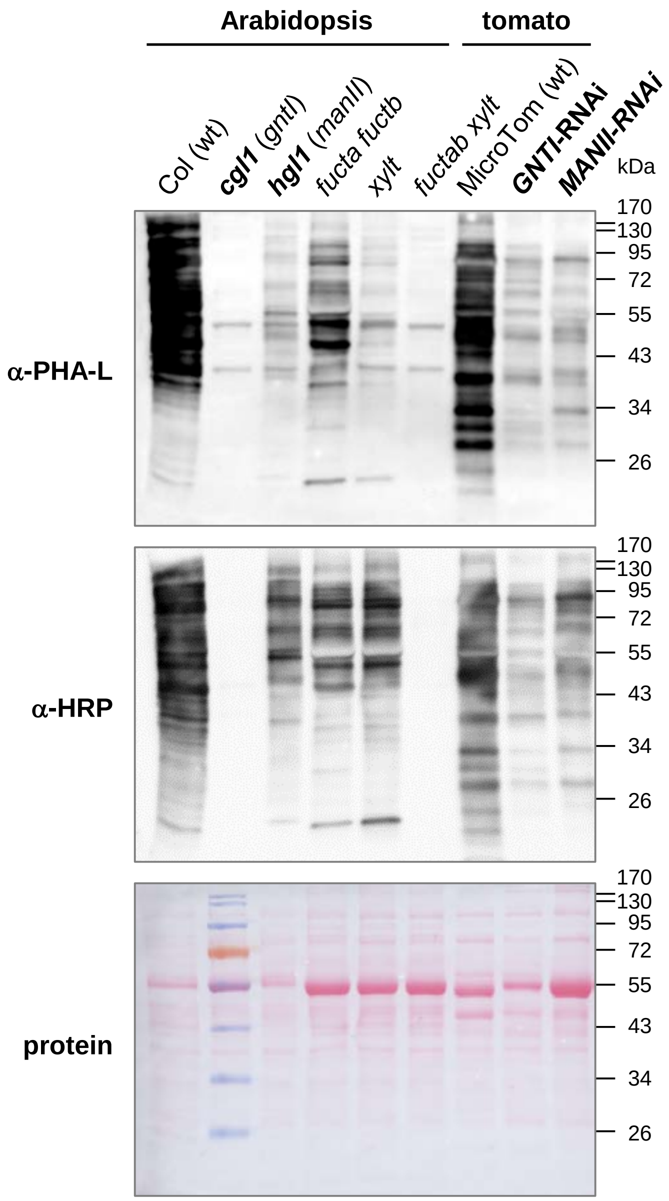

Figure S2. Comparison of Arabidopsis mutant to tomato RNAi plants.

Leaf extracts of Arabidopsis wild-type (Col) and different complex glycan mutants side-by-side with tomato Micro-Tom wild-type (wt), *GNTI*-RNAi (#20) and *MANII*-RNAi (#14). The blot was developed with two complex glycan (cgly) antisera. First with α-PHA-L (anti-Phytohemeagglutinin-L), and after stripping with α-HRP (anti-horseradish peroxidase), both vacuolar glycoproteins. Note that cgly detection of *MANII*-RNAi is comparable to the Arabidopsis *hgl1* (*manII* mutant). The Ponceau S-stained blot (protein) is shown as loading reference. Apparent molecular masses are indicated in kDa (PageRuler Prestained Protein Ladder, Fermentas).

Supports Figure 1

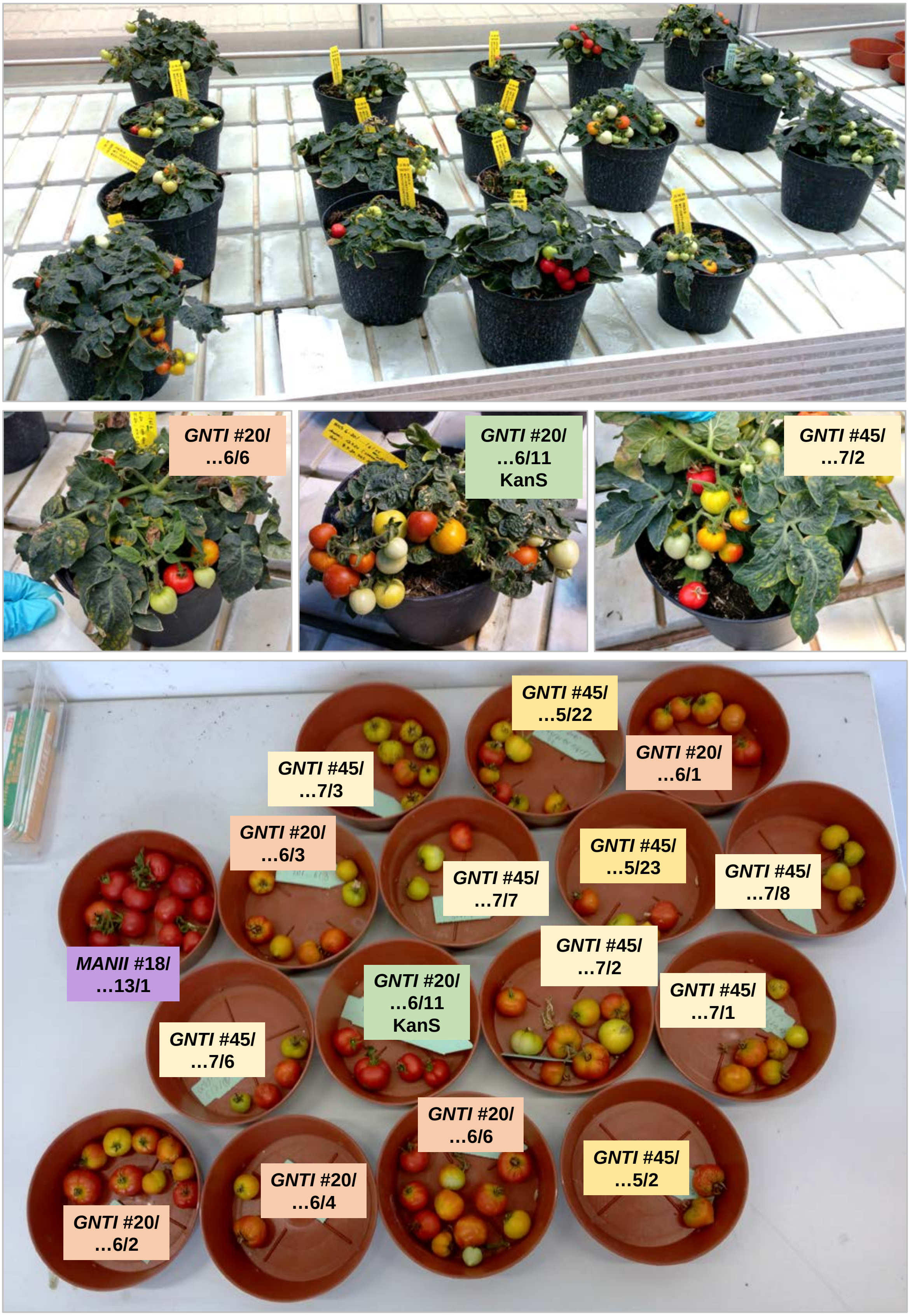

Figure S3A. Micro-Tom RNAi plants in the greenhouse and harvested fruits of different lines. Note that fruits of a Kanamycin-sensitve sibling in line #20 (green label) ripen regularly (pseudo wild-type).

Supports Figure 1

***MANII*-RNAi #14 (T4)**

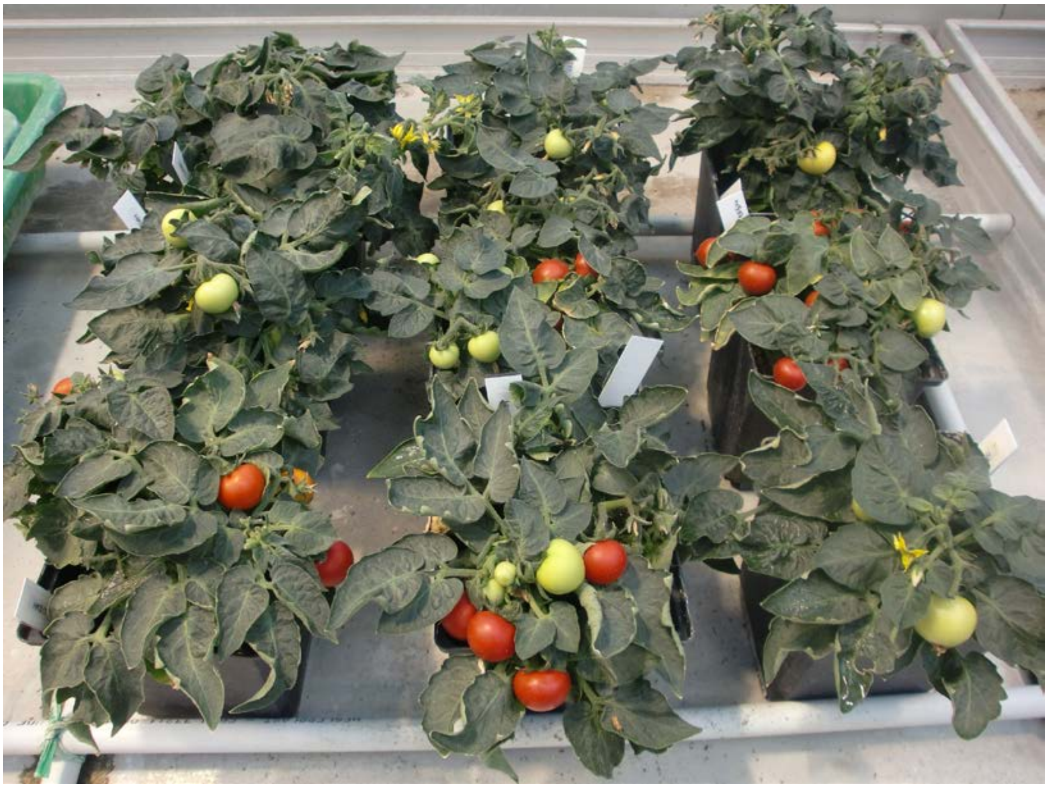

***MANII*-RNAi #18 (T1)**

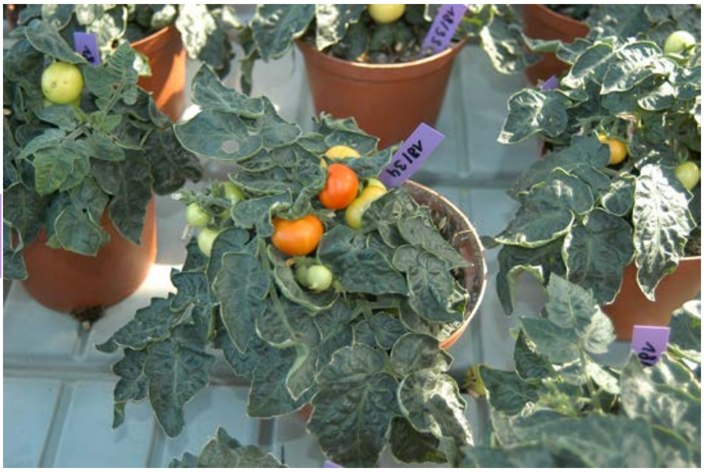

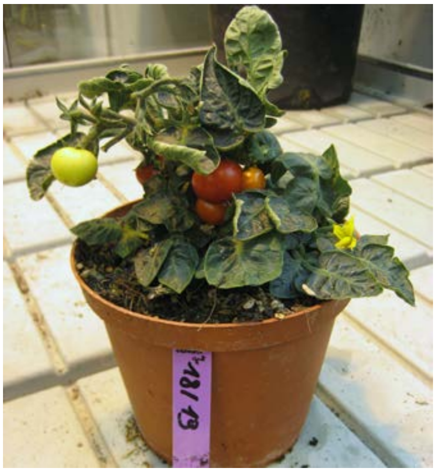

***MANII*-RNAi #11 (T1)**

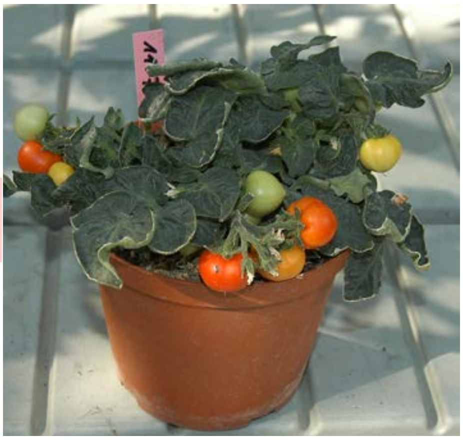

***MANII*-RNAi #15 (T1)**

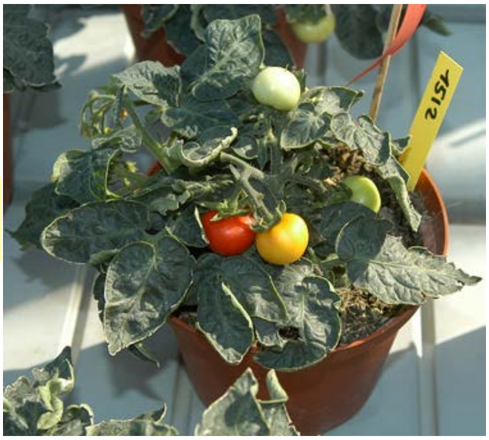

Figure S3B. Different *MANII*-RNAi lines with fruits in the greenhouse.

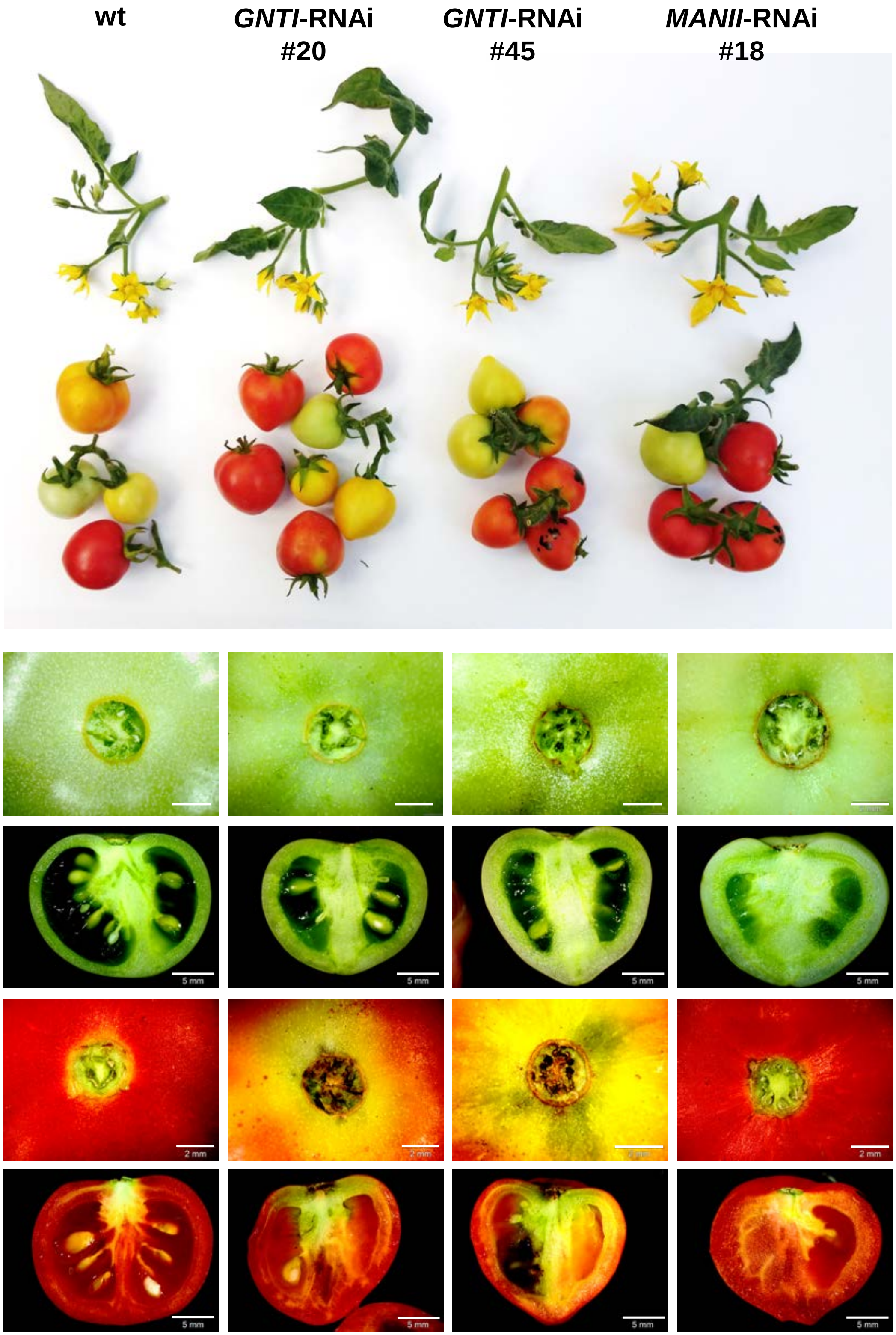

Figure S3C. Details of tomato Micro-Tom flowers, fruits, and fruit-attached regions.

Flowers and fruits were harvested from Micro-Tom wild-type (wt), two *GNTI*-RNAi lines and *MANII*-RNAi plants in the greenhouse. Details were photographed with a computer-assisted binocular. Bars represent 2 mm for fruit-attachment zones and 5 mm for fruit halves. Note the differences in size, seed number and pericarp thickness for fruits of the different genotypes.

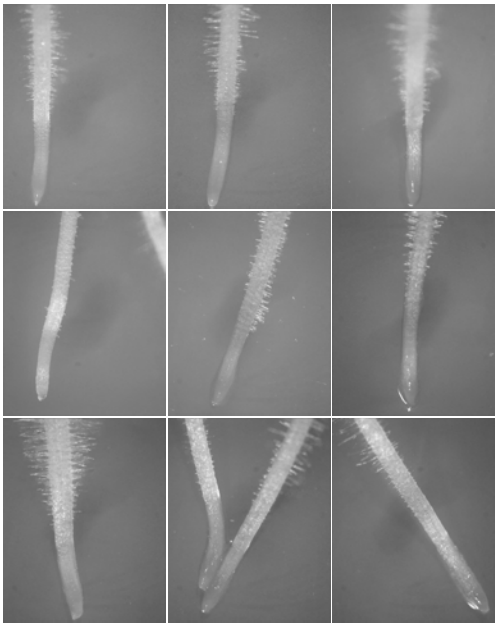

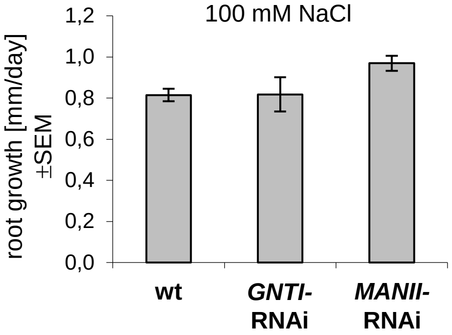

Figure S4. Root growth analysis of Micro-Tom seedlings.

Seeds of Micro-Tom wild-type (wt), *GNTI*-RNAi (#20) and two *MANII*-RNAi lines (#11, #14) were surface-sterilized and placed on MS agar containing 100 mM NaCl. To determine the increment of root growth, seedlings were photographed after 3 and 7 days of vertical growth in a climate chamber (long day regime). The diagram represents growth per day (in mm) ± SEM (standard error of the mean). wt, n=10; *GNTI*-RNAi, n=7; *MANII*-RNAi, n=14.

Supports Figure 2

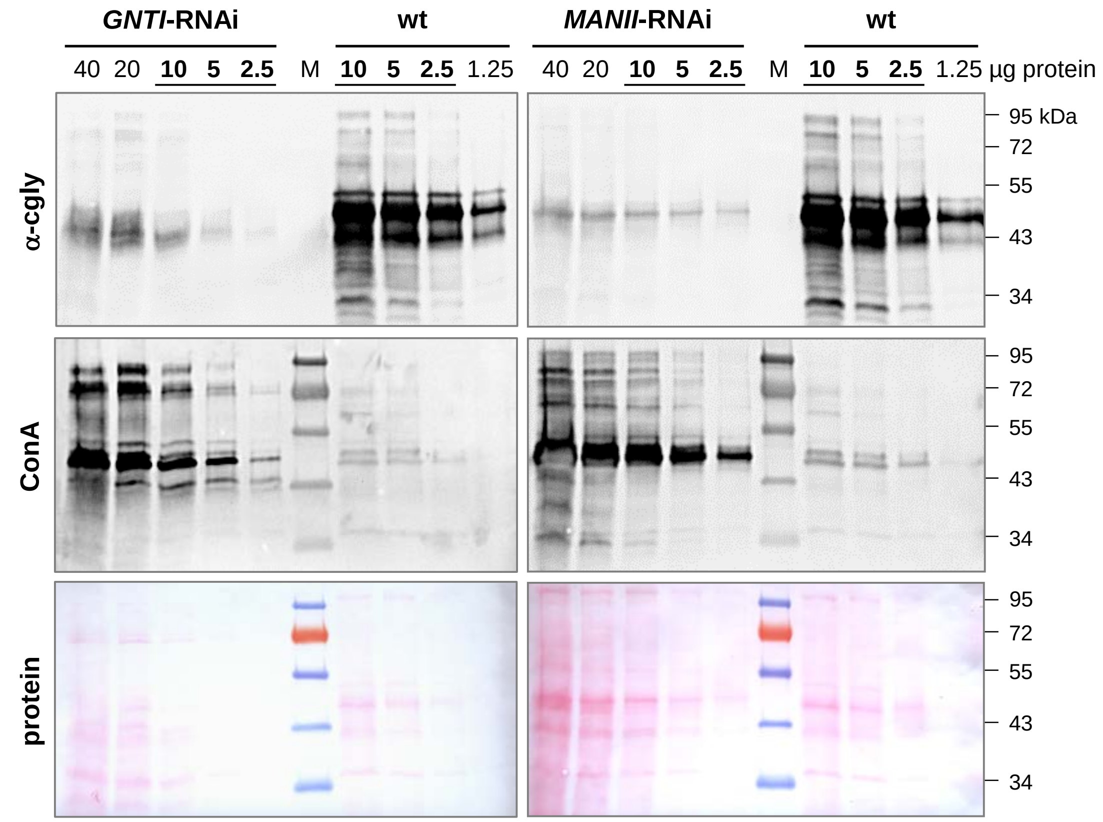

Figure S5. Extent of complex glycan reduction in fruits of Micro-Tom RNAi plants.

Protein amounts were determined with the Bradford assay in fruit extracts of comparable ripeness (Figure 2, grey stars) and serially diluted prior to immunoblot analysis. After complex glycan detection (α-cgly with α-PHA-L antiserum, top) the blot was stripped and developed with the lectin Concanavalin A (ConA) that binds to terminal mannose residues. Note that both *GNTI*-RNAi (#20) and *MANII*-RNAi (#6) result in about 50-times reduced cgly and inversely increased mannose detection. The Ponceau S-stained blots (protein) are shown as loading reference. Apparent molecular masses are indicated in kDa (PageRuler Prestained Protein Ladder, Fermentas).

Supports Figure 5

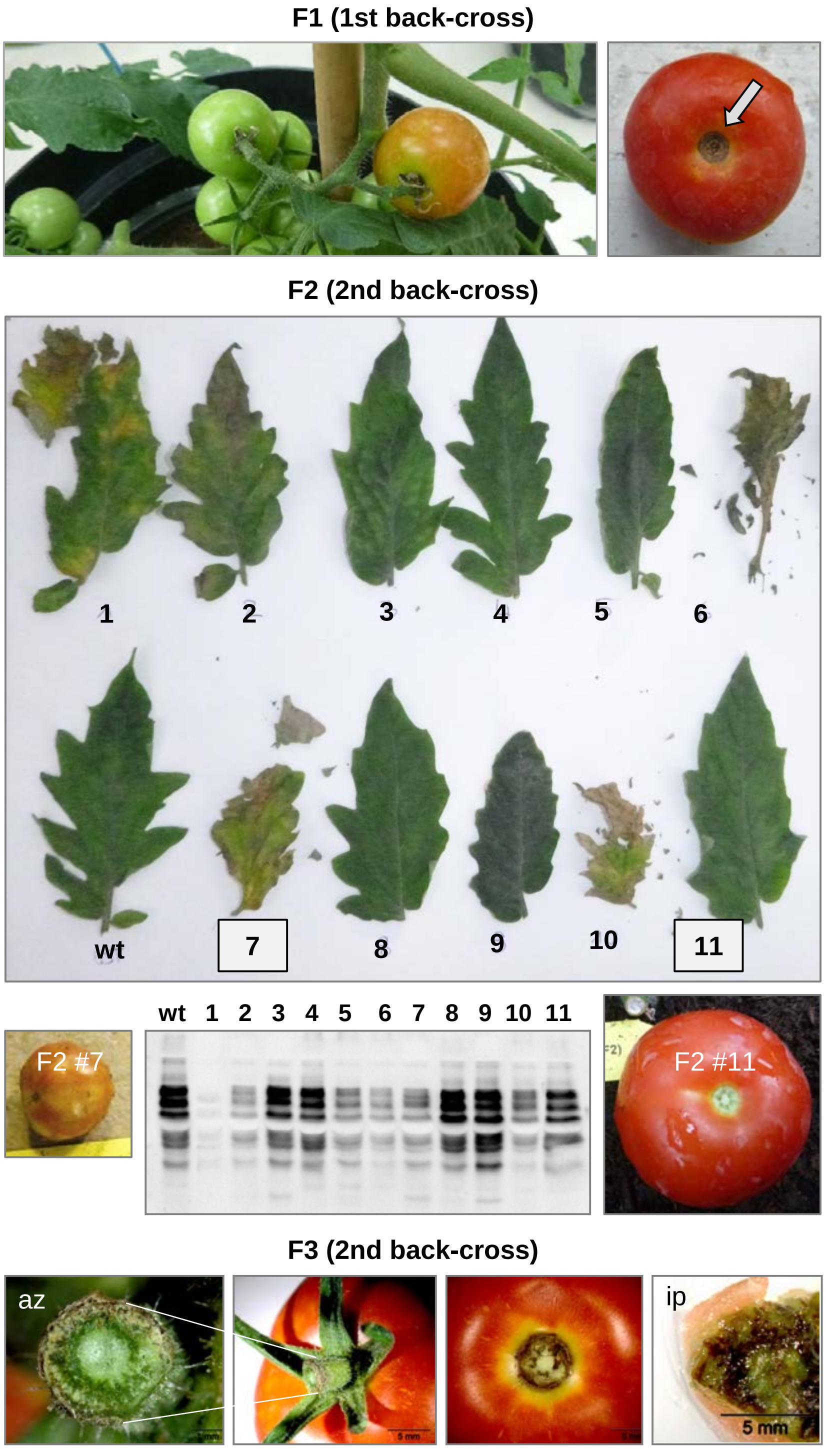

Figure S6. ***GNTI*-RNAi leaf and fruit phenotypes persist after back-crossing.**

Moneymaker wild-type served as mother plant for two consecutive back-crosses with Micro-Tom *GNTI*-RNAi line #20. F1 plants of the first back-cross developed incompletely ripe fruits with necrotic stalk-attached region (top right, arrow). F1 flowers were pollinated with Moneymaker wt (2nd back-cross). Resulting F2 plants showed segregating leaf and fruit phenotypes that correlated with complex glycan reduction (α-cgly blot, center), e.g. F2 #7 with necrotic leaf lesions (and signs of bacterial infection) produces small patchy fruits (left), but F2 #11 wild-type like leaves and fruits (right). Bottom, necrosis at the pedicel abscission zone (az) and fruit-attached region (ip, inner part) in the F3.

Supports Figure 5

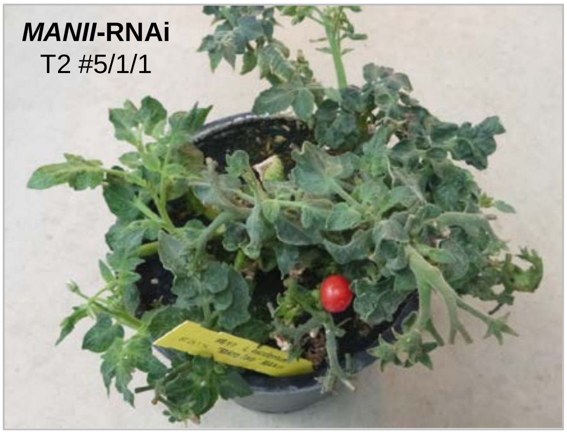

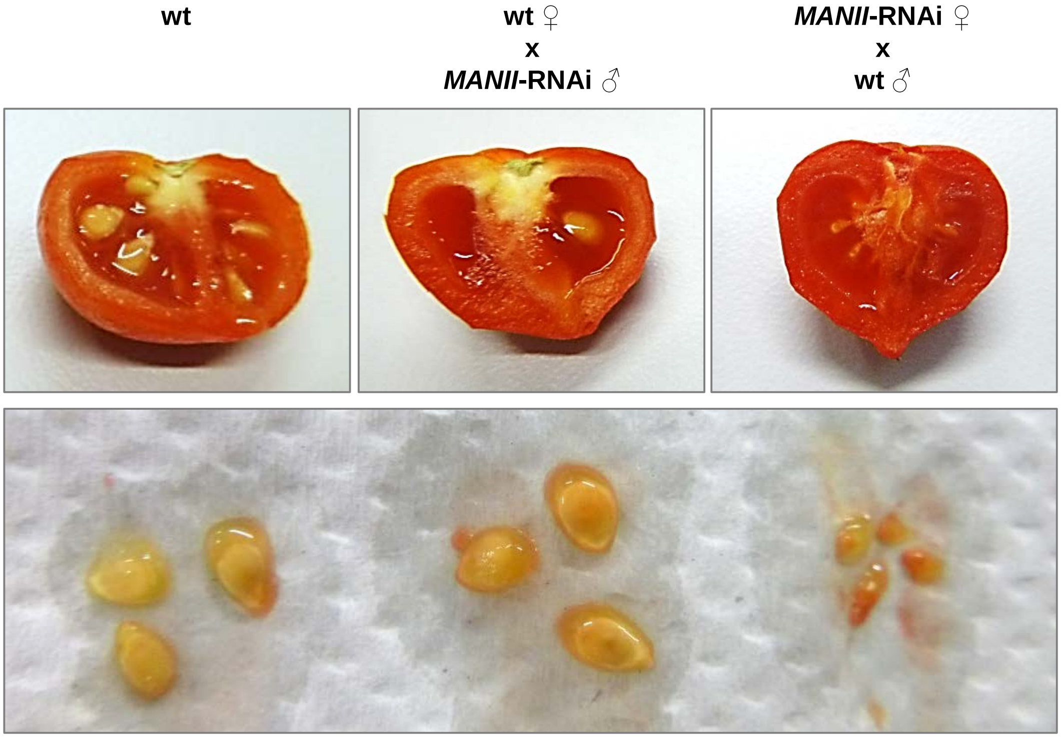

Figure S7. *MANII*-RNAi plants could not be propagated by back-crossing.

A *MANII*-RNAi transformant that produced only few seeds (#5, T2) was reciprocally crossed with Micro-Tom wild-type (wt). When *MANII*-RNAi served as pollen donor, normal looking seeds were obtained, but much smaller seeds when *MANII*-RNAi served as mother plant. Among the normal-looking seeds (of the cross with wt as mother plant) no resistant offspring was found, supporting fertilization problems of *MANII*-RNAi plants (selfed, compare Figure 5).

Supports Figure 5

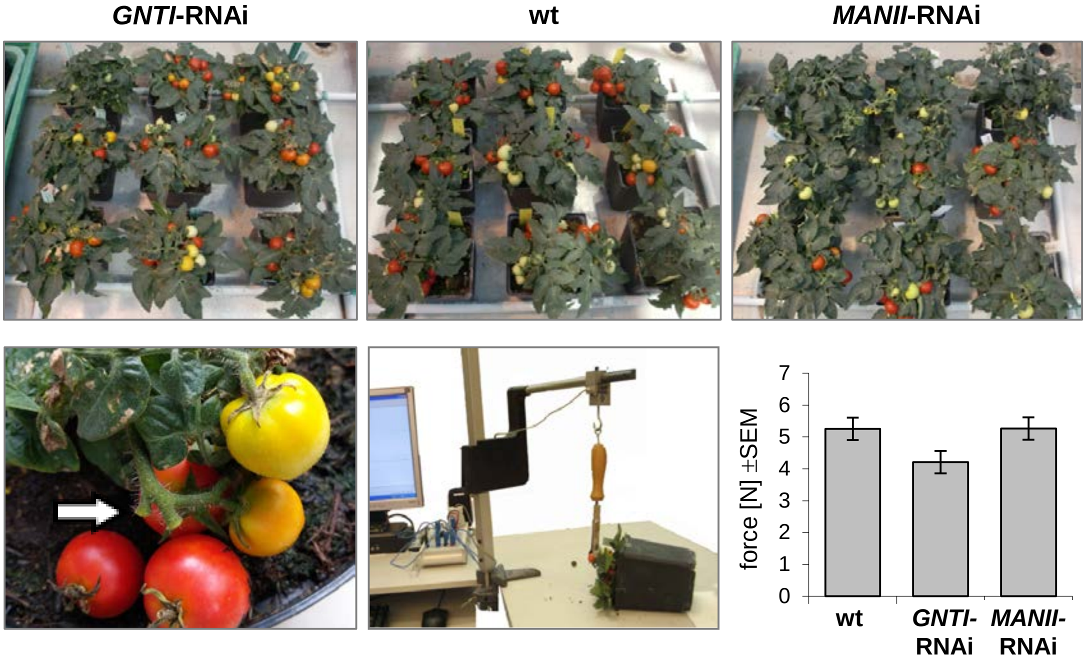

Figure S8. Fruits of *GNTI*-RNAi plants are released at lower pulling forces.

Micro-Tom plants in the greenhouse (3 months old) were used for measuring the force needed to pick a fruit. An example of early fruit drop (arrow) and experimental set-up with computer-assisted dynamometer is shown below. Red fruits of wild-type (wt), *GNTI*-RNAi (#20) and *MANII*-RNAi (#14) plants were pulled in longitudinal direction (relative to the stalk axis) and the resulting forces plotted, N, Newton; SEM, standard error of the mean (n = 18).

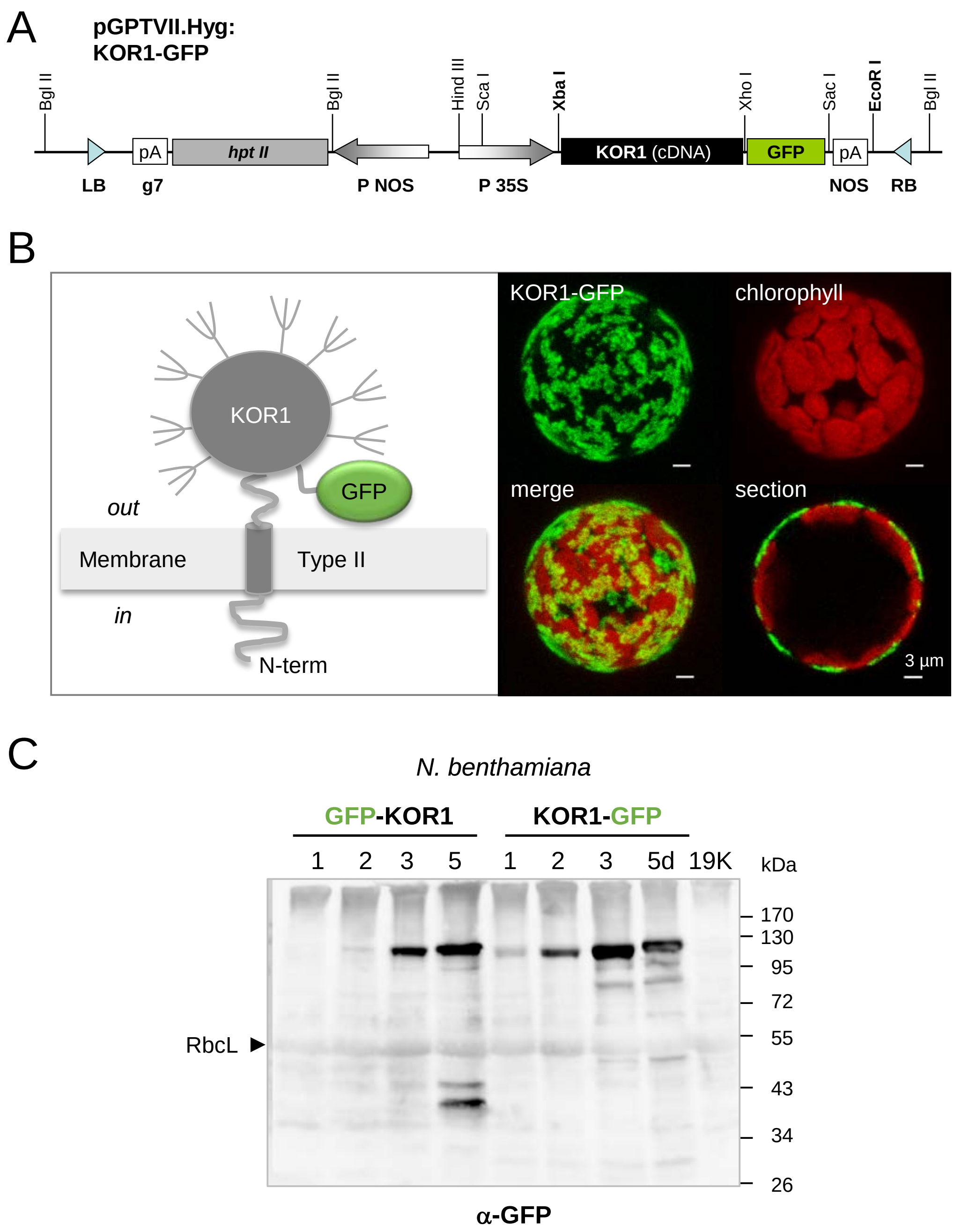

Figure S9. Generation of a binary KOR1-GFP reporter construct with labelled ectodomain.

A, Scheme of the binary KOR1-GFP construct (expression cassette, for abbreviations see Figure S1).

B, Type-II membrane topology with labeled *N*-glycosylated KOR1 ectodomain (left). The localization pattern of KOR1-GFP in tobacco protoplasts (right) resembles that of GFP-KOR1 (von Schaewen et al. 2015).

C, Agroinfiltration of *N. benthamiana* leaves with GFP-KOR1 and KOR1-GFP binary constructs. Immunoblot analysis of whole leaf extracts (Frank et al., 2008) with anti-GFP antbodies (α-GFP) show similar expression over a 5-day time course. Note the slightly different degradation products when GFP is fused to the *N*-glycosylated KOR1 ectodomain. 19K, co-expressed Agrobacterium silencing suppressor strain (control). RbcL, Large subunit of Ribulose-bisphosphate carboxylase/oxygenase. Apparent molecular masses are indicated in kDa (PageRuler Prestained Protein Ladder, Fermentas).
